# Noncollinear and nonlinear pulse propagation

**DOI:** 10.1038/s41598-018-32676-9

**Published:** 2018-09-25

**Authors:** Tomasz M. Kardaś, Yuriy Stepanenko, Czesław Radzewicz

**Affiliations:** 1 Institute of Physical Chemistry, Polish Academy of Sciences, Kasprzaka 44/52, 01-224 Warsaw, Poland; 2 Department of Physics, Institute of Experimental Physics, University of Warsaw, Pasteura 5, 02-093 Warsaw, Poland

## Abstract

A novel method for numerical modelling of noncollinear and nonlinear interaction of femtosecond laser pulses is presented. The method relies on a separate treatment of each of the interacting pulses by it’s own rotated unidirectional pulse propagation equation (UPPE). We show that our method enables accurate simulations of the interaction of pulses travelling at a mutual angle of up to 140°. The limit is imposed by the unidirectionality principal. Additionally, a novel tool facilitating the preparation of noncollinear propagation initial conditions - a 3D Fourier transform based rotation technique - is presented. The method is tested with several linear and nonlinear cases and, finally, four original results are presented: (i) interference of highly chirped pulses colliding at mutual angle of 120°, (ii) optical switching through cross-focusing of perpendicular beams (iii) a comparison between two fluorescence up-conversion processes in BBO with large angles between the input beams and (iv) a degenerate four-wave mixing experiment in a boxcar configuration.

## Introduction

One of the major achievements in modeling of ultrafast nonlinear processes is a framework for numerical simulation of second-order nonlinear processes based on only two assumptions: unidirectionality of propagation and paraxial approximation^1^. Even more fundamental approach, which drops the paraxial approximation and treats nonlinear polarization (and possibly free currents) in a general way can be derived from the vectorial Unidirectional Pulse Propagation Equation (UPPE)^2,3^. These models enable numerical simulation of nonlinear processes in the presence of frequency dependent diffraction, dispersion and spatial walk-off. They do not, however, immediately enable noncollinear beam propagation. Still, as they were the most accurate models avaiable they have been used for noncollinear simulations of Optical Parametric Chirped Pulse Amplifiers (OPCPA)^4–7^. Only a small range of noncollinearity angles has, however, been considered (bellow 1° ^7^ and several degrees–for tilted front pulses^6^) to preserve the accuracy of calculations.

Accurate as they are these models are also resource consuming. In the case of OPCPA the challenge comes from the fact that the interacting pulses are not only extremely broadband but can also be stretched up to nanosecond durations^8–10^, therefore, their representation requires huge numbers of points in the simulation grid. Consequently, the full 3D models are used only when the signal pulses are moderately chirped^6,7,11^. In other cases the numerical reality forces simplifications and, therefore, additional assumptions. For example, three dimensional simulations with neglected higher order dispersion and diffraction are often in use^12–14^. Another common practice applied to lower the memory requirements is to reduce the dimensionality. Thus two dimensional simulations with one of the Cartesian dimensions dropped^15–17^, sometimes with no diffraction and no walk-off included^18,19^, are used. Noncollinear OPA geometry is known to provide very large bandwidths^20–22^, the simpler–collinear propagation model is, however, often used to model its operation. A good example of this approach are the two dimensional models with cylindrical coordinates^23^. Beams in such simulations are intrinsically collinear. To account for noncollinearity ad hoc spatial overlap parameters are sometimes included^24,25^. Dispersionless models with spatial effects^26^ and finally, one dimensional simulations with dispersion treated up to the second order^27^, or even with no dispersion^28–30^ can also, without question, be of value in some applications.

The extra assumptions pointed out above are well justified when general qualitative results are of interest. However, a more accurate modeling is always tempting as it may reveal some subtle effects obscured by too crude approximations. Recently, some of us have shown that the use of a correct ab initio approach to nonlinear pulse interaction simulations applied to a design of a nonlinear optical device can result in the threefold efficiency increase with respect to the efficiency of previous solutions and an unprecedented reduction in the size of the device^31^. Therefore, a search for more accurate simulation methods and mathematical tools which could reduce the computational requirements for these methods is justified.

The need for a robust noncollinear simulation is even more pronounced in the case of broadband frequency mixing at large angles. The fluorescence up and down conversion processes with 10°–30° angles^32–34^ can serve as a good example. Apparently, to this day no propagation simulation approach to these problems have been attempted.

Yet another area of interest for noncollinear propagation is a variety of four-wave mixing (FWM) processes. Here degenerate and two-color resonant FWM^35–37^, Raman based^38–40^ and (with a proper model for the medium nonlinear polarization response) 2D-IR^41^ experiments can be considered. While these experiments are often performed in a noncollinear configuration the literature on appropriate propagation models is minimal or nonexistent.

Although, the direct solution of Maxwell equations with finite difference time domain (FDTD) methods can, in principle, be considered as an alternative to unidirectional methods, it suffers from signifcant flaws^42^. First, it requires resoling the whole simulation domain of interest (not only the vicinity of the pulse) with a resolution high enough for the features of the pulse itself to be resolved. Additionally, the time step is limited so to fit the spatial grid resolution (Courant condition). Therefore, a supercomputer cluster instead of a laptop and a time of several days instead of minutes is required per single simulation performed with FDTD. Also, FDTD presents complications when nonlinear effects (especially Raman scatteing) have to be implemented^43^. Finally, the accuracy of FDTD is much smaller than that of the spectral (unidirectional) methods^44^.

In the present paper we describe a novel method for numerical simulations of noncollinear pulse propagation and nonlinear interaction using just the unidirectionality approximation. In this method the angle between the interacting beams is limited only by the unidirectionality principle and, therefore can exceed 140° (depending on the beam size). The method relies on expressing the separate UPPE describing propagation of each of the beams in a single common spatial coordinates system. In this new system the interaction of pulses through nonlinear terms can easily be calculated while the rotated linear terms assure accurate propagation. Our approach is valid as long as the volumes of the spectral space used for representation of the particular interacting pulses with the same polarization do not overlap.

Our method enables simulation of noncollinearly propagating pulses. Some preparation of the input pulses (initial conditions) is, however, required. We, therefore, present a Fourier transform based 3D rotation procedure inspired by the image processing techniques^45^. This technique can be easily applied to rotations of a complex number based electric field. It is more than three orders of magnitude faster than the linear 3D interpolation even for relatively small grids. For large grids the Fourier rotation becomes an enabling tool as the rotation time can be reduced from days (as in case of interpolation) to minutes. It is also expected to be more accurate than the common interpolation techniques^45^.

To demonstrate the capabilities of our method, we present a number of linear and nonlinear propagation examples:

- linear interference of ultrashort pulses.
- optical switching through cross-focusing of beams crossing each other at a 90° angle.
- a fluorescence up-conversion in a BBO crystal where we compare type II, and type I configurations (with mutual angles between the interacting beams of 19°, and 27°, respectively).
- a degenerate four-wave mixing experiment in the boxcar configuration for which we observe spectral narrowing as the angle between the beams is increased.

## UPPE Framework

The most accurate unidirectional model of propagation in nonlinear media, derived from Maxwell equations with a single assumption of unidirectionality is the one based on the vectorial UPPE^2,3^:

1 $${\partial }_{z}{E}_{s}^{p}=i{k}_{z}^{p}{E}_{s}^{p}+{e}_{s}^{p}{{\bf{e}}}^{p}\frac{\tilde{\omega }}{2{\varepsilon }_{0}{c}^{2}{k}_{z}^{p}}(i\tilde{\omega }{{\bf{P}}}^{NL}-{\bf{j}}),\,s=x,y\,,$$

with ${{\bf{E}}}^{p}(\tilde{\omega },{k}_{x},{k}_{y},z)=({E}_{x}^{p},{E}_{y}^{p},{E}_{z}^{p})$–the complex electric field of polarization mode *p* represented in Fourier space with *ῶ*–the optical frequency and *k*_*x*_, *k*_*y*_–the spatial frequencies or wavevector $({{\bf{k}}}^{p}(\tilde{\omega },{k}_{x},{k}_{y},{k}_{z}^{p}))$ components. UPPE describes propagation of electric field along *z* or more specifically only the part of electric field propagating towards positive values of *z* as this is a unidirectional equation. The **e**^*p*^ = **E**^*p*^/|**E**^*p*^| is a unit polarization vector and |.| describes length of vector. The wavevector component along the propagation axis ${k}_{z}^{p}$ is related to the frequency, *k*_*x*_, *k*_*y*_ and refractive index *n*(*ῶ*, *k*_*x*_, *k*_*y*_) via the dispersion relation:

2 $${k}_{z}^{p}=\sqrt{{(\frac{\mathop{\omega }\limits^{ \sim }{n}_{p}(\mathop{\omega }\limits^{ \sim },{k}_{x},{k}_{y},{k}_{z}^{p})}{c})}^{2}-{\mathop{k}\limits^{ \sim }}_{x}^{2}-{\mathop{k}\limits^{ \sim }}_{y}^{2}},$$

For the case of birefringent materials Eq. (1). can be written down separately for each of the polarization modes i.e.: ordinary (*p* = *o*) and extraordinary (*e*) modes in uniaxial or slow (*s*) and fast (*f* ) modes in biaxial materials. In homogeneous medium representation of two modes is also required and one can select *p* = 1, 2 in this case. The dependence of ${k}_{z}^{p}$ on the optical frequency and the wavevector components (*k*_*x*_, *k*_*y*_) is responsible for dispersion and diffraction, respectively. The additional dependence of refractive index on *k*_*x*_, *k*_*y*_ present in biaxial materials is responsible for spatial walk-off (double refraction). All the above mentioned linear effects are treated exactly in UPPE. The **P**^*NL*^ = **P**^*NL*^(**E**, *ῶ*, *k*_*x*_, *k*_*y*_, *z*) and **j** = **j**(**E**, *ῶ*, *k*_*x*_, *k*_*y*_, *z*) are the nonlinear part of the polarization vector and the free current vector^46,47^. UPPE is solved for electric field components perpendicular to *z* axis. The missing *E*_*z*_ component of electric field vector required for calculation of **P**^*NL*^ and **j** can, however, be obtained from *E*_*x*_ and *E*_*y*_^2,48^.

From the numerical point of view it is convenient to use electric field envelope instead of **E**^*p*^:

3 $${\tilde{{\bf{E}}}}^{p}(t)={\tilde{{\bf{A}}}}^{p}(t){{\bf{e}}}^{-i{\omega }_{R}t}+c.c,$$

where *ω*_*R*_ is a certain reference frequency (usually the central frequency of the pulse). This substitution is equivalent to variable change (shift operation) in the Fourier space, the Fourier transform of Eq. (3) reads:

4 $${{\bf{E}}}^{p}(\tilde{\omega })={{\bf{A}}}^{p}(\tilde{\omega }-{\omega }_{R})={{\bf{A}}}^{p}(\omega ),$$

where *ω* = *ῶ* − *ω*_*R*_ is the detuning from the pulse central frequency.

## Idea of the Method

**Figure 1:**
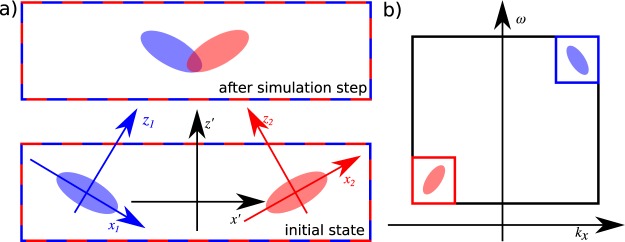
The scheme of noncolinear simulation. The interacting pulses have to be represented in overlapping spatio-temporal grids (red and blue rectangles) (**a**). Thus, the two UPPE describing propagation of the “blue” pulse along *z*_1_ axis and “red” pulse along *z*_2_, have to be represented in a new coordinates system *x*′ − *y*′ − *z*′. In this system the nonlinear terms can be calculated without interpolation. At the same time the spectral volumes (red and blue rectangles in **b**) corresponding to the two pulses cannot overlap (unless the polarization of the two are orthogonal) so that none of the plane wave modes is represented in more than one grid.

The central idea of our work is to represent each of the interacting pulses in a separate discrete cuboid grid and write a rotated UPPE for each of the pulses separately (see Fig. 1(a).). The grids’ nodes have to overlap exactly in the spatio-temporal domain so that the nonlinear mixing terms can be calculated. Therefore, each of the interacting beams, propagating along axes *z*_*l*_ (with *l* = 1, 2...) in a *x*_*l*_ − *y*_*l*_ − *z*_*l*_ system, will require solution of its own UPPE represented in a new coordinate system *x*′ − *y*′ − *z*′:

5 $${\partial }_{z^{\prime} }{A^{\prime} }_{s}^{p}=i\frac{1}{{c}_{\theta }}({K^{\prime} }^{p}-\eta {c}_{\theta }\kappa ^{\prime} -{s}_{\theta }{c}_{\varphi }{k^{\prime} }_{x}-{s}_{\theta }{s}_{\varphi }{k^{\prime} }_{y}){A^{\prime} }_{s}^{p}+\frac{1}{{c}_{\theta }}{Q}^{^{\prime} p}{P}^{^{\prime} NL,p}.$$

where the first term describes the linear and the second term nonlinear effects, respectively. The definitions of variables used in Eq. (5), together with the equation derivation are given in Methods section.

**Figure 2:**
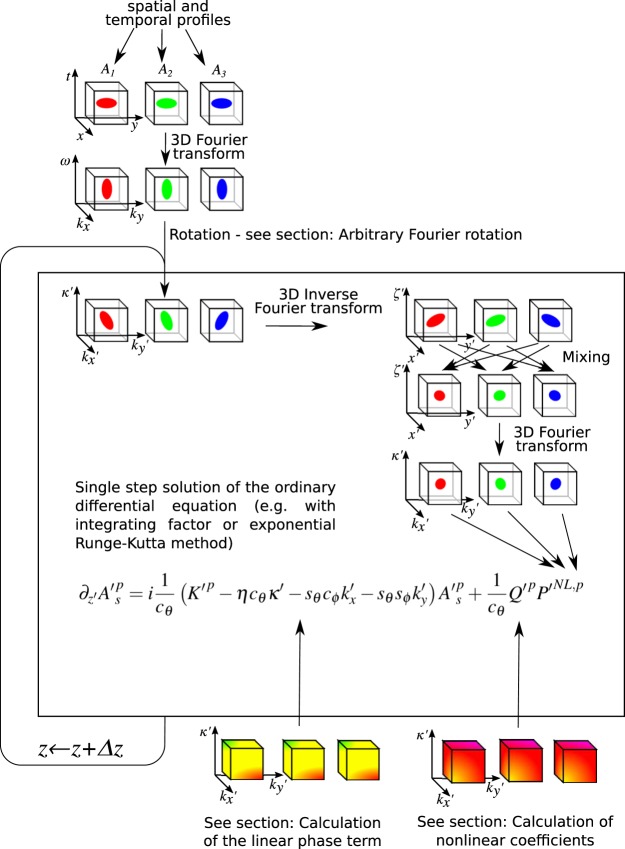
(5)efeq:UPPErot is solved by an ordinary differential equation solver in the ${k^{\prime} }_{x}$ − ${k^{\prime} }_{y}$ − *κ*′ space and in each step of the simulation the nonlinear term is computed in the *x*′ − *y*′ − *t*′ space.

**Figure 3:**
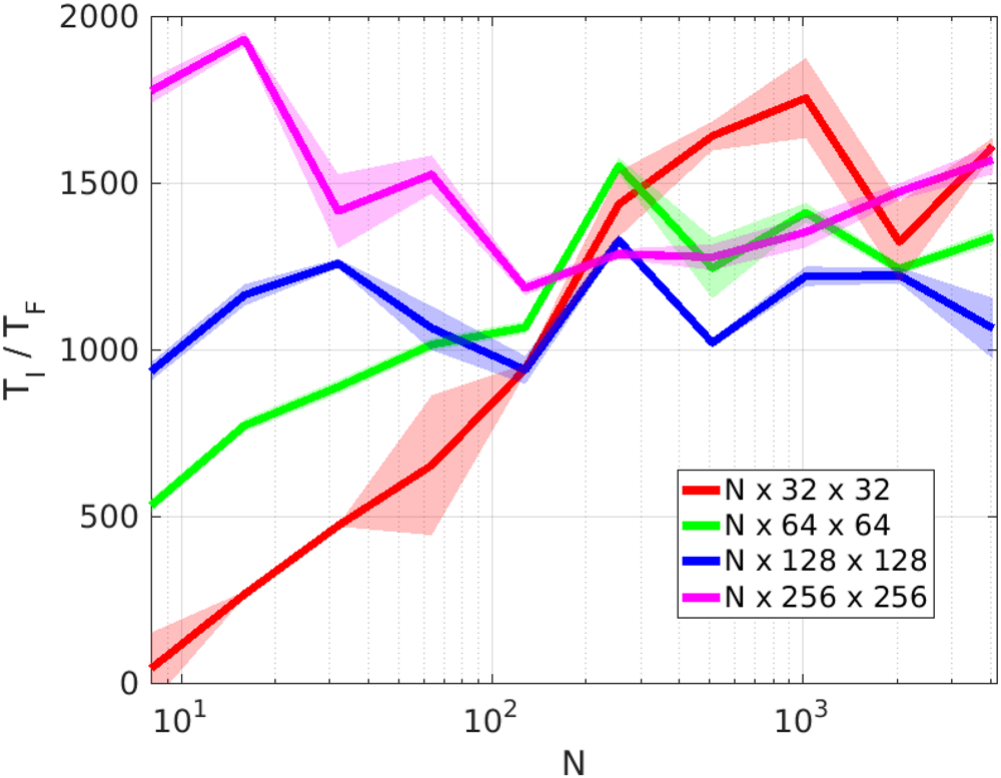
Ratio of the time required by interpolation (MATLAB’s griddata function, *T*_*I*_) and the Fourier transform based rotation (*T*_*F*_). For different grid sizes. Grid sizes are indicated in the legend.

**Figure 4:**
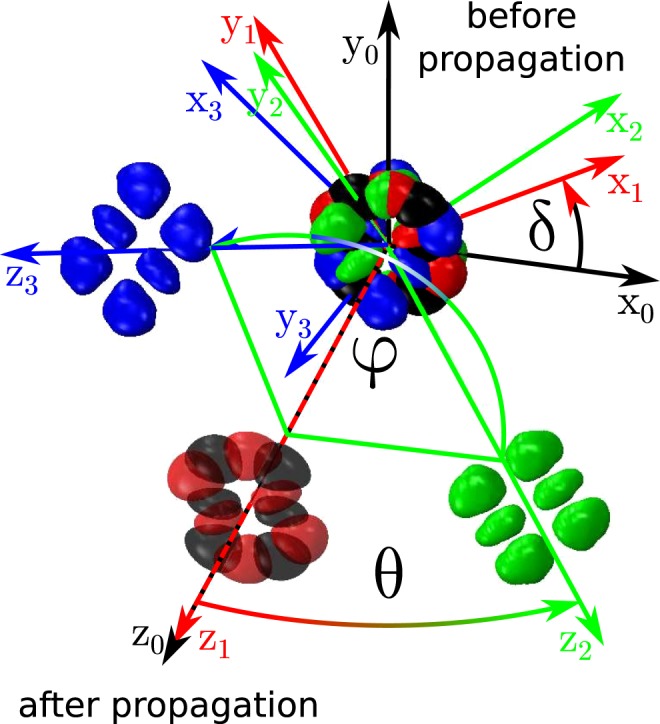
A pulse with Gaussian temporal profile (20 fs FWHM) and a Hermite-Gaussian beam profile (TEM 2, 1, *w*_0_ = 15 *μ*m) propagated collinearly (black) and noncollinearly (blue) with respect to the simulation axes *x*_0_, *y*_0_, *z*_0_. Three rotations: by *δ* = 30° around *z*_0_ (to *x*_1_, *y*_1_, *z*_1_), *θ* = 5° around *y*_0_ (to *x*_2_, *y*_2_, *z*_2_) and *φ* = 135° around *z*_0_ (to *x*_3_, *y*_3_, *z*_3_) are performed. After each rotation the pulse is propagated over a 200 *μ*m distance in fused silica.

The flow chart of the simulation is presented in Fig. 2. for an example of three wave mixing. First, preparation of the initial conditions: the electric field envelopes (*A*_*i*_, *i* = 1, 2, 3) in the *t*′ − *x*′ − *y*′ space is required. Often it is interesting to simulate propagation of complicated shape of electric field. It can involve Hermite and Laguerre spatial modes as well as secant, sinc, super-Gaussian or just arbitrary temporal shape known from SPIDER or FROG measurement of an experimental pulse. While, discretization of such shapes in arbitrarily rotated coordinates (*t*′ − *x*′ − *y*′) is difficult it is easy in non rotated grids. It is, therefore, of essence to find a procedure for arbitrary rotation of electric field within discretized grids. Moreover, this procedure is required at the end of simulation for back rotation of the fields and retrieval of output temporal and spatial profiles. In the Methods’ “Arbitrary Fourier rotation” section we present such a procedure based on Fourier transform and shear operations. This method can be faster than interpolation by as much as 3 orders of magnitude (see Fig. 3). An example of rotated pulse propagation is presented in Fig. 4.

**Figure 5:**
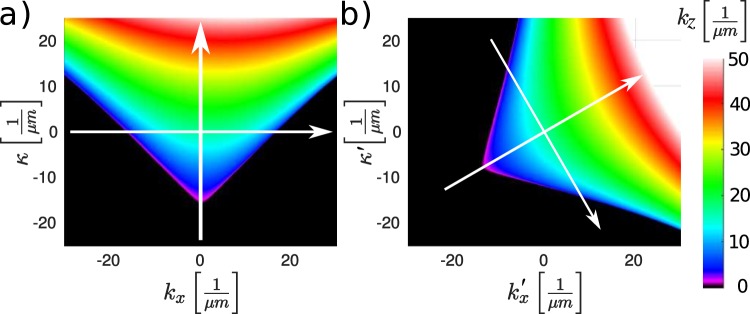
Linear phase factor (*k*_*z*_(*κ*, *k*_*x*_)) calculated for extraordinary pulse propagating in YVO_4_ at an angle of 48° with respect to the crystal’s optical axis (**a**). The white arrows point along the *κ* and *k*_*x*_ axes. The phase factor calculated for a pulse propagating at 60° with respect to the simulation’s *z* axis, but with the same direction with respect to medium as in a (**b**).

In the process of simulation the linear phase term (*k*_*z*_ from Eq. (1). or *K*′^*p*^ from Eq. (5).) has to be represented in *x*′ − *y*′ − *z*′ system for each of the beams. To this end a third coordinates system–the crystal optical axes *x*^*C*^ − *y*^*C*^ − *z*^*C*^ system has to be considered. For example see Fig. 5, where: *κ* = *ω n*(*ω*_*R*_, 0, 0)/*c* = *ω n*_*R*_/*c* is the normalized detuning from the reference frequency. Here ${k}_{z}^{e}$ was calculated through Eq. (2). for highly birefringent YVO_4_ crystal for the angle between *z* and *z*^*C*^ axes of 48° (maximum walk-off). The dispersion comes from slight deviation from linear growth of the phase along the vertical axis in Fig. 5(a). The diffraction manifests itself through circle like behaviour of *k*_*z*_ along the horizontal axis. And finally the presence of walk-off reveals itself in slight asymmetry of the colour pattern around the vertical axis. There are no propagating wave solutions for ${k}_{x}^{2}+{k}_{y}^{2} > {(n\tilde{\omega }/c)}^{2}$ (black color). In birefringent media the calculation of ${k}_{z}^{p}$ requires an iterative procedure involving multiple transitions between the three coordinate systems. This procedure is described in the “Calculation of the linear phase term” section. Calculation of the nonlinear polarization also require adequate vectorial treatment which is given in the section “Calculation of nonlinear coefficients”.

In each step of the simulation the nonlinear polarization is calculated in the *x*′ − *y*′ − *t*′ space–this requires inverse 3D Fourier transformation and back transformation of the mixing result to the ${k^{\prime} }_{x}$ − ${k^{\prime} }_{y}$ − *κ*′ space. Finally, Eq. 5. is solved by an ordinary differential equation solver e.g.: integrating factor or exponential Runge-Kutta method.

Our approach is valid as long as the volumes of the spectral space (*k*_*x*_ − *k*_*y*_ − *ω*) used for representation of particular interacting pulses with the same polarization do not overlap (see Fig. 1(b).). Note that this limitation is not as strict as the one imposed on the collinear propagation where the optical spectra of the interacting pulses cannot overlap^49^. In our case the extremely broad pulses can be used as long as the sum of their divergence angles is smaller then the noncolinearity angle by a certain factor (1.5 for the separation of 3*σ* for Gaussian beams). We have verified that this condition is easily fulfilled for a standard NOPA setup.

## Method’s Accuracy

First the results of forward propagation (*θ* = 0°) for beam sizes above 10 *μ*m, for which the paraxial approximation is valid, have been compared with with SNLO^50^ and Hussar software^31^. The results were found to be in perfect agreement. Then the results of propagation for various values of *θ* have been compared to results of the forward propagation. Both linear and nonlinear (SHG) propagation through 5 mm BBO crystal have been tested for various beam sizes (3–300 *μ*m) and pulse durations (3–300 fs). The linear propagation tests have also been performed for constant width of 10 *μ*m and pulse durations down to 1.15 fs (1.4 cycle) and for various width (*w*_0_ ≤ 1 *μ*m) and constant duration of 10 fs in 1 mm of fused silica. The nonlinear propagation requires multistep solver, thus, the Integrating Factor Runge-Kutta 4, 5 Dormand-Prince method (IFRK45)^51^ with adaptive step-size control^52^ have been used. The linear problem can be solved in a single propagation step as in this case: ${E}_{s}^{p}(z+{\rm{\Delta }}z)={E}_{s}^{p}(z){e}^{i{k}_{z}^{p}{\rm{\Delta }}z}$ exactly. Thus, the Exponential Euler method (EE)^53^, which does not subdivide the steps into smaller parts was used. Both: Integrating Factor and Exponential methods are specially designed for problem with large linear therm.

In case of rotated UPPE method the ultimate limitation appears when pulse contains components that would propagate in the negative *z*′ axis direction. These components cannot be propagated with a unidirectional model and, thus, the error must grow near the 90° limit. In fact we have verified that for *θ* ≤ 60° (corresponding to a 120° mutual angle for a two beam case) the error in beam waist, pulse duration and energy was bellow 3·10^−3^ for the single step linear cases (for pulses longer then 1.15 fs the errors are bellow 10^−5^). For the nonlinear case the error depends on the step size. We have found, however, that, for a given distance, it can be easily reduced down to 10^−4^ for reasonable step sizes–a multiple of the light wavelength (grid size 1024(t) × 1024(x)). The detail description of the tests of the method is present in the supplementary material.

## Examples

For each of the examples, two *z*-marching schemes were used–IFRK45 and an EE equipped with Richardson extrapolation^52^ for automatic step size selection. The step sizes were selected automatically so that the relative errors bellow 10^−6^ in each step were assured. The sizes and densities of the grids were increased to a point at which relative differences in parameters resulting from consecutive simulations: energies, beam waists and pulse durations were below 10^−3^ (usually much less). Additionally, the spectral, spatial and temporal profiles were also compared visually. This convergence has occurred for grid sizes that enabled simulation on a 16 GB RAM laptop computer. The results have been, however, confirmed with simulations using larger grids run on a 64 GB RAM PC.

### Pulse interference

**Figure 6:**
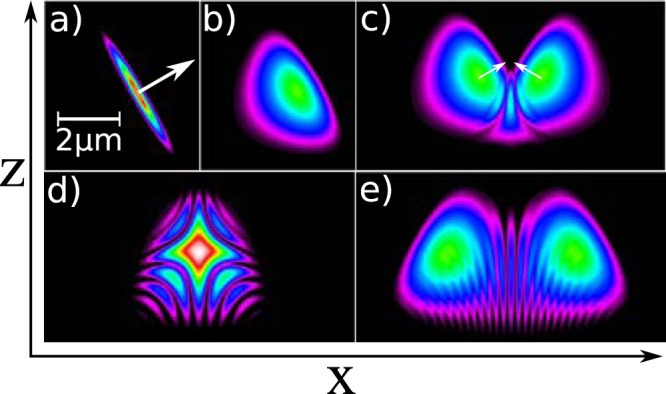
The 3 fs (FWHM) and 3 *μ*m (waist) pulse before (**a**) and after (**b**) propagation through 20 *μ*m of ZnSe. The same pulse interfering with a similar pulse approaching the crossing point at mutual angle of 120° at three locations: 20 *μ*m before (**c**), 0 *μ*m (**d**) and 40 *μ*m after (**e**) the approximate crossing point (see supplementary movie for complete interference pattern evolution).

The noncollinear but linear propagation has a potential application for solving interference problems. Here an example interference pattern for two pulses crossing each other at a mutual angle of 120° is visualized. The pulses are created by propagation of two 3 fs FWHM Gaussian pulses with 3 *μ*m waist and central wavelength of 800 nm through 20 *μ*m of ZnSe. A “top view” of the initial pulse and the result of propagation are presented in Fig. 6(a,b) respectively. Three stages of interference are presented in Fig. 6(c–e), the Supplementary Movie 1. also presents the evolution of the interference pattern during pulse propagation.

### Optical Switching through XPM

The cross-phase modulation and resulting cross-focusing has been considered as a candidate mechanism for optical switching in dielectric media^54–58^. It has been obtained eperimentally in gases^59^ where the band of a supercontinuum of a probe beam was controlled through interaction with the pump. In this work apart of phase-modulation the Raman effect played an important role. Beam direction switching through attraction of beams traveling in the same direction has been studied for coaxial^54^, and displaced beams^55^. It has been studied extensively in fibers^56^ also for supercontinuum generation purposes^57^ and in fiber gratings^58^. Cross-focusing of coaxial beams have also been extensively studied in the context of plasma physics^60–62^.

**Figure 7:**
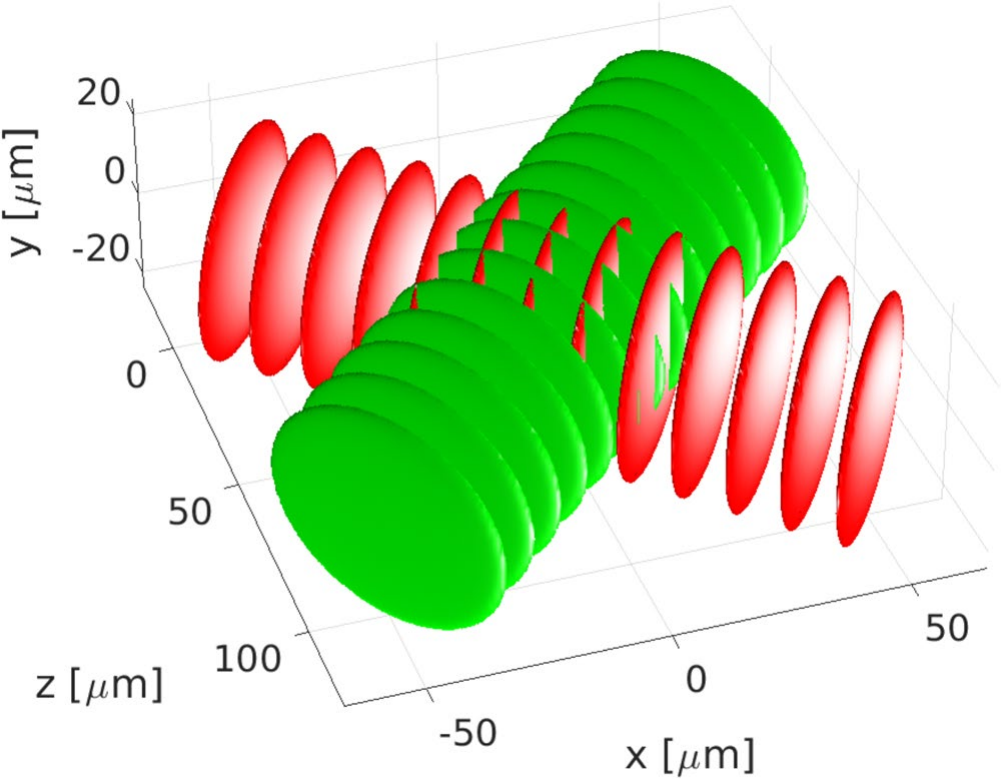
Cross-Phase Modulation configuration.

In the present section a cross-focusing effect occurring between two beams with polarizations along the *y* axis (see Fig. 7.) crossed at 90° is studied. The nonlinear polarization for SPM and XPM can in this case be written as:

6 $${\tilde{P}}_{NL}^{\mathrm{1/2}} \sim {n}_{2}(|{\tilde{A}}_{\mathrm{1/2}}{|}^{2}+\frac{2}{3}|{\tilde{A}}_{\mathrm{2/1}}{|}^{2}){\tilde{A}}_{\mathrm{1/2}},\,{\rm{with}}\,{n}_{2}={e}_{y}{\chi }_{yyyy}^{\mathrm{(3)}}{e}_{y}{e}_{y}{e}_{y}.$$

where indices 1 and 2 refer to the interacting pulses.

**Figure 8:**
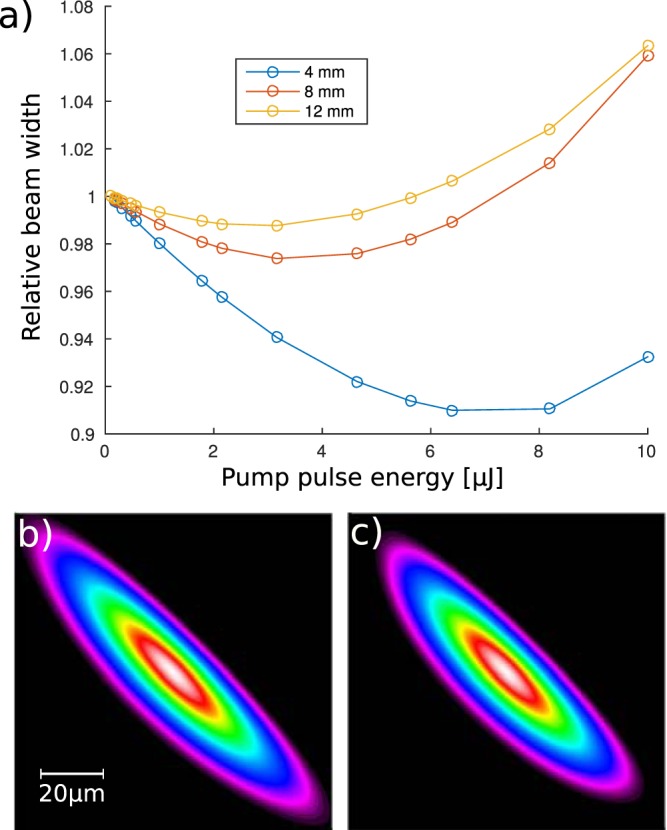
Change of the beam width caused by interaction with a pump beam for different pump pulse energies at three locations away from the interaction point (**a**). Top view of the probe pulse 4 mm away from the point of interaction with the pump pulse for pump energy of 0.1 *μJ* (**b**) and 6.4 *μJ* (**c**). Pulses are traveling towards top right corner of the figure.

**Figure 9:**
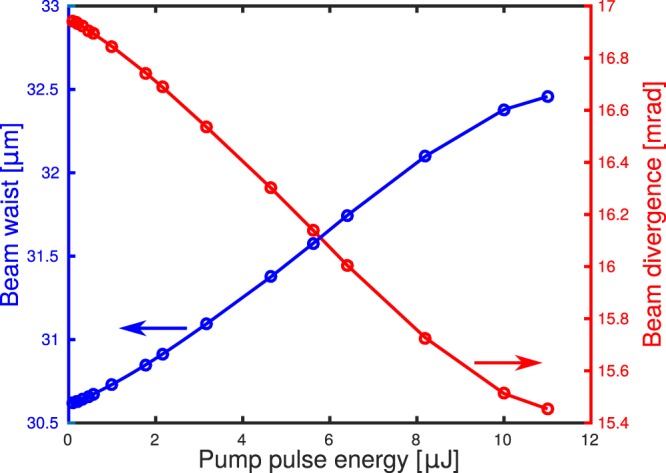
Dependence of beam waist and beam divergence on the pump pulse energy in the Cross-Phase Modulation simulation.

The simulation of cross-focusing of two Gaussian pulses (10 fs FWHM and 33 *μ*m of waist and wavelength of 800 nm) in 50 *μ*m-thick fused silica plate (${n}_{2}=3\times {10}^{-20}\frac{{{\rm{m}}}^{{\rm{2}}}}{{\rm{W}}}$) is performed. The energy of one of the pulses (the pump) is varied while the energy of the second pulse (probe) is set to a constant value of 1 *μ*J. Figure 8(a) presents the relative change of the beam width at three points located 4, 8 and 12 mm away from the pulse crossing point. Apparently even for such small interaction length (equal to the beam size) a significant change in size by around 10% can be observed 4 mm from the crossing point for the pump energy of 6.4 *μ*J. The “top view” spatial profile of the probe beam at this location for 0.1 and 6.4 *μ*J energy of the pump pulse is presented in Fig. 8(b,c) respectively. Figure 9 presents the dependence of the beam waist (spot-size in the beam focus) and the change in the beam divergence for different pump pulse energies.

If the probe pulse was focused into another medium the supercontinuum generation could be optically switched on as in the presence of the pump pulse the spot size change in the beam focus is as high as by 7% giving a 11% increase in the intensity. For energies of the pump pulse above 10 *μ*J the pump becomes strongly affected by SPM resulting in a distorted probe pulse.

### Highly noncollinear up-conversion

**Figure 10:**
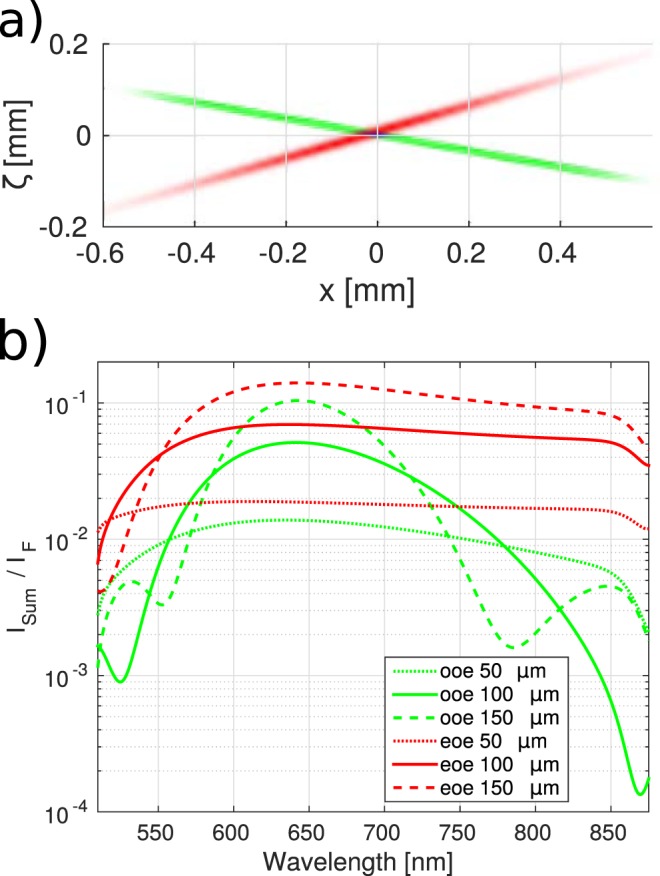
Spatial overlap of the interacting pulses (**a**), fluorescence (green), gate (red), up-converted light (blue). The normalized spectral power density of the up-converted fluorescence light transposed into the fluorescence wavelengths range (**b**). The type I (green) and type II (red) conversion is depicted for 50, 100 and 150 *μm* of propagation in BBO.

In this section we will present the first ever 3D numerical simulation of the fluorescence up-conversion process under high mutual angles of the fluorescence and gate beams. The search for optimal conditions for efficient and broadband up-conversion in spectroscopy is a subject of a live debate lasting at least since the begining of the century^32–34,63^. Here, broadband type I (ooe) and II (eoe) up-conversion processes in BBO crystal are compared. The fluorescence in range from 500 nm to 900 nm and the gate beam at 1020 nm were selected. For type I interaction the following geometry is assumed: *θ*_*G*_ = 31.9°, *θ*_*F*_ = 58° and *φ* = 90° (*d*_eff_ = 1.4 pm/V) with indexes *G* and *F* corresponding to the gate and fluorescence beams, respectively. For type II: *θ*_*G*_ = 34.3°, *θ*_*F*_ = 53.4° and *φ* = 0° (*d*_eff_ = 1.18 pm/V). The mutual beam angles are 26° and 19°, respectively. The gate pulse with a Gaussian temporal profile (100 fs FWHM), energy of 11 *μ*J in a Gaussian beam was selected to simulate conditions of the experimental setup at IPC PAS^63^. The beam waist was assumed to be *w*_0_ = 0.5 mm. The fluorescence was modelled by a pulse with a super-Gaussian (flat top) spectrum ranging from 510 nm to 865 nm. The fluorescence pulse was chirped with 50 fs^2^ to the duration of 100 fs (FWHM). Figure 10(a) presents the three interacting pulses (see also Supplementary Movie 2). The up-converted light arises where the gate pulse overlaps with the fluorescence. In the real life experiment the gating pulse has to be tilted to achieve high temporal resolution of the setup. In the present paper pulses without tilt are used to highlight basic properties of new propagation simulation method.

The spectral power density of the up-converted signal (*I*_*Sum*_) normalized to the fluorescence spectral power density (*I*_*F*_) for three thicknesses of the BBO crystal and for the two phase-matching types are presented in Fig. 10(b). The inverse of this quantity is the “correction factor”^34^ for retrieving fluorescence from the up-converted light. The type II phase-matching delivers both: more efficiency and a broad spectrum up-conversion. This confirms its superiority over type I phase-matching for 1020 nm gate and is consistent with results obtained experimentally for 1340 nm gate^34^. Note, that for the 150 *μm* crystal the quantum up-conversion efficiency exceeds 0.1.

### Degenerate four-wave mixing

Degenerate four-wave mixing between pulses at 1550 nm in a box car configuration is considered in the present section. Two narrowband pump pulses (super-Gaussian temporal profile of the sixth order with FWHM of 1 ps) are sent into a highly nonlinear 5 mm ZnSe crystal (*n*_2_ ≈ 10^−18^ m^2^/W^64^) together with a broadband (super-Gaussian spectral profile of the third order with FWHM of around 100 nm) signal pulse stretched to around 1.1 ps through introduction of 16 000 fs^2^ of dispersion.

**Figure 11:**
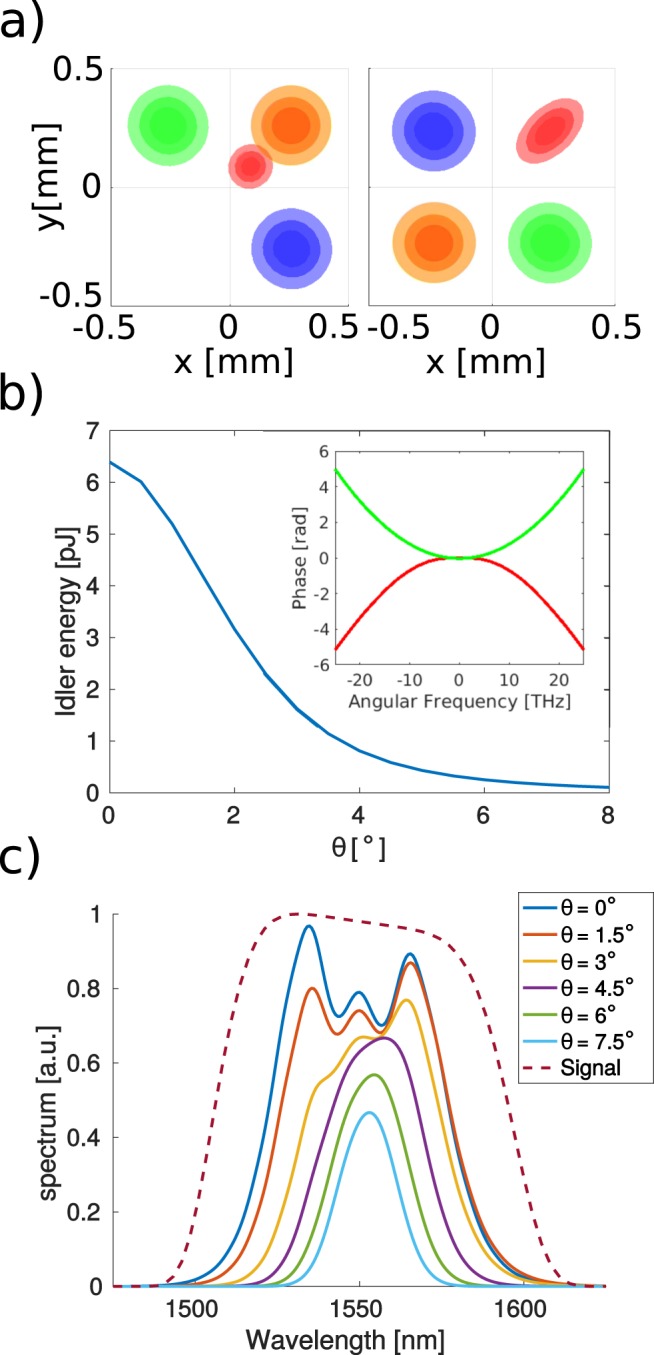
Intensity distributions of the pump (blue and green), signal (orange) and idler (red) at the input (left) and output (right) of the 5 mm ZnSe crystal (**a**). Some idler is already generated at the input of the crystal in the area where beam tails overlap. The idler pulse energy as a function of the noncollinearity angle (**b**), phases of the signal (green) and idler (red) pulses are displayed in the inset. Spectra of the idler pulse for different values of the noncollinearity angle (**c**).

In the case of collinear propagation the different spectral parts of the signal pulse constantly overlap with the two long pump pulses as all the pulses have the same group velocities. Through FWM a fourth (idler) pulse is created. The idler pulse is broadband and its phase is similar to that of the signal pulse with, however, an opposite sign (see inset on Fig. 11(b)). This feature of FWM is well known and it is some times used for phase imprinting especially for purposes of “time lens” realization^65,66^.

When the box car configuration is used the three input beams cross at the center of the crystal. At this point different temporal parts of the pulses overlap at different times. Fronts of the pulses interact, as they reach the crossing point (where the spatial overlap occurs) first. Then the temporal centers and finally tails of the pulses have the chance to interact.

The situation suggests that, as well as in the collinear situation, all the frequencies will be amplified in a similar way. This is, however, not the case because the idler pulse is generated mostly in the area where the other three beams overlap. Figure 11(a) presents the pulse intensities at the beginning of the sample, the idler pulse (red) appears in the overlap area of the pump (green and blue) and the signal (orange) pulses. At the beginning the front parts of the pulses have the highest overlap, therefore, the red frequencies from the signal pulse are amplified the most. Later on, the center frequency, and finally, the “bluest” frequency of the signal pulse is amplified as the center parts and tails of the pulses interact, respectively. The central parts of the pulses interact for the longest time, therefore, the spectrum of the idler pulse becomes narrower with respect to the collinear case (see Fig. 11(c)). Moreover, the generated idler pulse walks-off spatially in a constant manner as it has its direction defined trough the phase-matching relation (see also the Supplementary Movie 3). As a consequence the idler pulse becomes spatially stretched (see Fig. 11(a)) and tilted–with the pulse front perpendicular to the average propagation direction of all the pulses. Obviously, the overall conversion efficiency of the process will diminish when the angle between the beams is increased, as the interaction length is decreased (see Fig. 11(b) for the energy of the generated idler pulse). A simple non-resonant FWM was considered in our model. A premise, however, exists that the boxcar configuration should be used with caution also in resonant spectroscopic experiments. Proper simulation for particular problems can be performed when the 3D propagation model is combined with an appropriate model for the medium (spectroscopic sample) nonlinear response.

We estimate that performing an analogous simulation with FDTD would require ~3500 times more RAM (above 1 TB) and ~85 times more computational steps, which can translate into days of simulation instead of 1 minute with the current approach.

## Conclusions

We have presented a novel method for numerical simulation of noncollinear pulse propagation and nonlinear interaction. We have found that the method works properly even for the mutual angles between the propagation directions of the interacting pulses as high as 140°, it is limited by unidirectional approximation. The techniques have been tested on linear and nonlinear propagation examples.

We have also presented a novel method for arbitrary 3D roation of the complex electric field. Our method, in comparison to interpolation presents a speed-up on a level of three orders of magnitude.

Our simulations shown that optical switching is possible through cross-focusing even for very short interaction lengths i.e. even in the case of perpendicular pulse routes. We have shown that the type II phase-matching delivers both: more efficiency and a broad spectrum up-conversion and, thus, it is a preferable method for fluorescence up-conversion in BBO. Finally, we have shown that increase of noncolinearity angle in degenerate four-wave mixing experiment can lead to spectral narrowing of the generated signal pulse.

## Methods

### UPPE in rotated coordinates

It is convenient to use three Euler angles and to represent the actual pulse direction as a result of three consecutive rotations: around *z*′ axis by *δ* (*R*_*z*′_(*δ*)), around *y*′ axis by *θ* (*R*_*y*′_(*θ*)) and again around *z*′ by *ϕ* (*R*_*z*′_(*ϕ*)). The “normalized frequency” (*κ*) and time ($\zeta =-\,\frac{c\tau }{{n}_{R}}$) are used to simplify notation. Apart of the rotation, in the propagation model, the moving reference frame would be welcome^67,68^. The latter also requires the change of variables with “local normalized time” *ζ*′ = *ζ* − *ηz*, where *η* = *c*/*n*_*R*_*v* and *v* is the velocity of the window. The change of variables is thus defined by, first: rotation and then transition to a moving reference frame:

7 $$(\begin{array}{c}x^{\prime} \\ y^{\prime} \\ z^{\prime} \\ \zeta ^{\prime} \end{array})=(\begin{array}{cccc}1 & 0 & 0 & 0\\ 0 & 1 & 0 & 0\\ 0 & 0 & 1 & 0\\ 0 & 0 & -\eta  & 1\end{array})(\begin{array}{cccc}{c}_{\varphi }{c}_{\theta }{c}_{\delta }-{s}_{\varphi }{s}_{\delta } & -{c}_{\varphi }{c}_{\theta }{s}_{\delta }-{c}_{\delta }{s}_{\varphi } & {c}_{\varphi }{s}_{\theta } & 0\\ {s}_{\varphi }{c}_{\theta }{c}_{\delta }+{c}_{\varphi }{s}_{\delta } & -{s}_{\varphi }{c}_{\theta }{s}_{\delta }+{c}_{\delta }{c}_{\varphi } & {s}_{\varphi }{s}_{\theta } & 0\\ -{c}_{\delta }{s}_{\theta } & {s}_{\delta }{s}_{\theta } & {c}_{\theta } & 0\\ 0 & 0 & 0 & 1\end{array})(\begin{array}{c}x\\ y\\ z\\ \zeta \end{array}),$$

or:

8 $$(\begin{array}{c}x^{\prime} \\ y^{\prime} \\ z^{\prime} \\ \zeta ^{\prime} \end{array})=(\begin{array}{cccc}{c}_{\varphi }{c}_{\theta }{c}_{\delta }-{s}_{\varphi }{s}_{\delta } & -{c}_{\varphi }{c}_{\theta }{s}_{\delta }-{c}_{\delta }{s}_{\varphi } & {c}_{\varphi }{s}_{\theta } & 0\\ {s}_{\varphi }{c}_{\theta }{c}_{\delta }+{c}_{\varphi }{s}_{\delta } & -{s}_{\varphi }{c}_{\theta }{s}_{\delta }+{c}_{\delta }{c}_{\varphi } & {s}_{\varphi }{s}_{\theta } & 0\\ -{c}_{\delta }{s}_{\theta } & {s}_{\delta }{s}_{\theta } & {c}_{\theta } & 0\\ {c}_{\delta }{s}_{\theta }\eta  & -\eta {s}_{\delta }{s}_{\theta } & -\eta {c}_{\theta } & 1\end{array})(\begin{array}{c}x\\ y\\ z\\ \zeta \end{array}).$$

where a shorted notation: *s*_*α*_ = sin*α*, *c*_*α*_ = cos*α* have been used. With this in mind the derivative ∂_*z*_ form UPPE can be expressed as:

9 $${\partial }_{z}={\partial }_{z}x^{\prime} {\partial }_{x^{\prime} }+{\partial }_{z}y^{\prime} {\partial }_{y^{\prime} }+{\partial }_{z}z^{\prime} {\partial }_{z^{\prime} }+{\partial }_{z}\zeta ^{\prime} {\partial }_{\zeta ^{\prime} }={c}_{\varphi }{s}_{\theta }{\partial }_{x^{\prime} }+{s}_{\varphi }{s}_{\theta }{\partial }_{y^{\prime} }+{c}_{\theta }{\partial }_{z^{\prime} }-\eta {c}_{\theta }{\partial }_{\zeta ^{\prime} }$$

which, after Fourier transform (∂_*ζ*′_ → −*iκ*′, ∂_*x*′/*y*′_ → *ik*_*x*′/*y*′_) becomes:

10 $${\partial }_{z}=i{c}_{\varphi }{s}_{\theta }{k}_{x^{\prime} }+i{s}_{\varphi }{s}_{\theta }{k}_{y^{\prime} }+{c}_{\theta }{\partial }_{z^{\prime} }+i\eta {c}_{\theta }\kappa ^{\prime} $$

To simplify the notation in this section UPPE (Eq. (1)) is written in the contracted form:

11 $${\partial }_{z}{A}_{s}^{p}=i{K}^{p}{A}_{s}^{p}+i{Q}^{p}{P}^{NL,p}$$

with:

12 $${K}^{p}(\kappa ,{k}_{x},{k}_{y})={k}_{z}^{p}(\frac{c\kappa }{{n}_{R}}+{\omega }_{R},{k}_{x},{k}_{y}),\,{Q}^{p}(\kappa ,{k}_{x},{k}_{y})=\frac{{(\frac{c\kappa }{{n}_{R}}+{\omega }_{R})}^{2}}{2{\varepsilon }_{0}{c}^{2}{k}_{z}^{p}(\frac{c\kappa }{{n}_{R}}+{\omega }_{R},{k}_{x},{k}_{y})}{e}_{s}^{p},$$

and *P*^*NL*,*p*^ = **e**^*p*^**P**^*NL*^ where the envelope defined by Eq. (3). was used.

With the above described variable change rotated UPPE yields:

13 $${\partial }_{z^{\prime} }{A^{\prime} }_{s}^{p}=i\frac{1}{{c}_{\theta }}({K^{\prime} }^{p}-\eta {c}_{\theta }\kappa ^{\prime} -{s}_{\theta }{c}_{\varphi }{k^{\prime} }_{x}-{s}_{\theta }{s}_{\varphi }{k^{\prime} }_{y}){A^{\prime} }_{s}^{p}+\frac{1}{{c}_{\theta }}{Q^{\prime} }^{p}{P^{\prime} }^{NL,p}$$

where ${K^{\prime} }^{p}/{Q^{\prime} }^{p}/{A^{\prime} }_{s}^{p}/{P^{\prime} }^{NL,p}(\kappa ^{\prime} ,{k}_{x^{\prime} },{k}_{y^{\prime} })$ represent rotated ${K}^{p}/{Q}^{p}/{A}_{s}^{p}/{P}_{NL,p}$. The procedure for calculation of these variables is descried in next sections.

### Transitions between coordinate systems

In multi-pulse propagation simulations it is convenient to set the direction of one of the beams with respect to the simulation coordinates system and to define the direction of all the pulses with respect to the (often birefringent) crystal. We therefore give expressions for transitions between the three coordinate systems:

14 $$({{\bf{x}}}_{B},{{\bf{y}}}_{B},{{\bf{z}}}_{B})={R}_{BS}\,({{\bf{x}}}_{S},{{\bf{y}}}_{S},{{\bf{z}}}_{S}),$$

15 $$({{\bf{x}}}_{C},{{\bf{y}}}_{C},{{\bf{z}}}_{C})={R}_{CS}\,({{\bf{x}}}_{S},{{\bf{y}}}_{S},{{\bf{z}}}_{S}),$$

16 $$({{\bf{x}}}_{B},{{\bf{y}}}_{B},{{\bf{z}}}_{B})={R}_{BC}\,{\boldsymbol{(}}{{\bf{x}}}_{C},{{\bf{y}}}_{C},{{\bf{z}}}_{C}),$$

where (***x***_*B*_, ***y***_*B*_, ***z***_*B*_), (***x***_*C*_, ***y***_*C*_, ***z***_*C*_), (***x***_*S*_, ***y***_*S*_, ***z***_*S*_) represents the system of the beam, crystal and simulation, respectively.

If the orientation of the beam with respect to the simulation coordinate system is known and defined by the angles *ϕ*_*B*_, *θ*_*B*_ and *δ*_*B*_, then:

17 $${R}_{BS}={R}_{{z}_{S}}({\varphi }_{B}){R}_{{y}_{S}}({\theta }_{B}){R}_{{z}_{S}}({\delta }_{B}),$$

where *R*_*a*_(*α* describes rotation about axis *a* by an angle *α*. If the orientation of the crystal with respect to the simulation coordinate system is known and defined by the angles *ϕ*_*C*_, *θ*_*C*_ and *δ*_*C*_, then:

18 $${R}_{CS}={R}_{{z}_{S}}({\varphi }_{C}){R}_{{y}_{S}}({\theta }_{C}){R}_{{z}_{S}}({\delta }_{C}),$$

and:

19 $${R}_{BC}={R}_{CS}^{-1}{R}_{BS}$$

Note that *R*_*BS*_ and *R*_*CS*_ represent rotations around axes of a system (**x**_*S*_, **y**_*S*_, **z**_*S*_) in which the very matrices are represented (***x***_*S*_, ***y***_*S*_, ***z***_*S*_ form a identity matrix), thus, they have a simple classical form, similar to that of the upper left part of second transformation matrix in Eq. (7).

If, on the other hand, the crystal orientation with respect to the simulation frame (*ϕ*_*C*_, *θ*_*C*_ and *δ*_*C*_) and the beam orientation with respect to the crystal orientation (*ϕ*, *θ* and *δ*) is known, then:

20 $${R}_{BS}={R}_{{z}_{C}}(\varphi ){R}_{{y}_{C}}(\theta ){R}_{{z}_{C}}(\delta ){R}_{CS}.$$

Note that the matrix ${R}_{{z}_{C}}(\varphi ){R}_{{y}_{C}}(\theta ){R}_{{z}_{C}}(\delta )$ has a much more complicated form. This is a fact, as (**x**_*C*_, **y**_*C*_, **z**_*C*_) are not in general parallel to the unit vectors defining the simulation coordinate systems. These vectors are, however, available as the columns of *R*_*CS*_ matrix and the form of the matrix defining a rotation about an arbitrary vector can be found in reference^69^.

Finally, if beam propagation direction (*ϕ*_*B*_, *θ*_*B*_, *δ*_*B*_) and the material orientation with respect to the beam (*ϕ*, *θ*, *δ*) are known:

21 $${R}_{CS}={({R}_{{z}_{B}}(\varphi ){R}_{{y}_{B}}(\theta )R{z}_{B}(\delta ))}^{-1}{R}_{BS},$$

and the directions of (**x**_*B*_, **y**_*B*_, **z**_*B*_) are the columns of *R*_*BS*_ matrix. The matrix *R*_*BS*_ and angles *ϕ*, *θ*, *δ* are required for calculation of the linear propagation phase term *K*'^*p*^ as will be shown in the next section. The angles *ϕ*, *θ*, *δ* can be obtained from *R*_*BC*_^70^.

### Calculation of the linear phase term

The iterative procedure of calculating ${k}_{z}^{p}$ for forward pulse propagation has been described before^1^. In case of noncollinear propagation the procedure differs slightly.

First for each set of discrete simulation coordinates (*κ*′, *k*_*x*′_, *k*_*y*′_) a corresponding set of coordinates from the beam reference frame (*κ*, *k*_*x*_, *k*_*y*_) is calculated through rotation Eq. (14). This operation creates 3 matrices of *κ*, *k*_*x*_ and *k*_*y*_ values, each numbered by *κ*′, *k*_*x*′_ and *k*_*y*′_.

Then, matrices of *θ*(*κ*, *k*_*x*_, *k*_*y*_) and *ϕ*(*κ*, *k*_*x*_, *k*_*y*_)–angles defining the direction of propagation of each of the plane waves (defined by *κ*, *k*_*x*_, *k*_*y*_) with respect to the crystal orientation are initialized to given values defining the general beam direction (*θ*_0_ and *ϕ*_0_). Again *θ* and *ϕ* are matrices numbered by *κ*′, *k*_*x*′_ and *k*_*y*′_, the corresponding values of *κ*, *k*_*x*_ and *k*_*y*_ are, however, known from the previous step.

At this point the iterative method starts. Based on the values of *θ* and *ϕ* the values of refractive index *n*_*p*_ = *n*_*p*_(*ῶ*, *θ*, *ϕ*) are calculated (where $\tilde{\omega }=\frac{c\kappa }{{n}_{R}}+{\omega }_{R}$ is the optical frequency). The refractive index can be calculated from Sellmeier formula and properties of refractive index ellipsoid^71^. Finally the length of the wavevector ($|{{\bf{k}}}^{p}|=\frac{\tilde{\omega }{n}_{p}}{c}$) and the linear phase term (${k}_{z}^{p}=\sqrt{|{{\bf{k}}}^{p}{|}^{2}-{k}_{x}^{2}-{k}_{y}^{2}}$) can be calculated.

Now a set of wavevectors: $({k}_{x},{k}_{y},{k}_{z}^{p})$ for all the plane waves describing the pulse in the beam reference frame is known. With the inverse of rotation Eq. (16) it is transformed to the crystal coordinate system $({k}_{x}^{C},{k}_{y}^{C},{k}_{z}^{pC})$.

The values of *θ* and *ϕ* can now be updated through:

22 $$\theta =\arctan (\frac{\sqrt{{k}_{x}^{C2}+{k}_{y}^{C2}}}{{k}_{z}^{pC}}),\,\varphi =\arctan (\frac{{k}_{y}^{C}}{{k}_{x}^{C}})$$

and the next iteration can be started.

The iteration can be stopped when the change of ${k}_{z}^{p}$ value becomes negligible (around 20 iterations are sufficient to obtain relative accuracy of 10^−13^ for standard birefringent materials).

### Calculation of nonlinear coefficients

The coefficient matrix *Q*′^*p*^(*κ*′, *k*_*x*′_,*k*_*y*′_) can already be calculated from *K*^*p*^ and Eq. (12). We will describe the treatment of the nonlinear coefficient *P*′^*NL*,*p*^ on the example of second order nonlinearity.

Note first that in absence on nonlinearity birefringent media electric field vector of a particular mode (*o*, *e*, *s*, *f*) is uniquely defined by *ῶ*, *k*_*x*_ and *k*_*y*_. The procedure for finding **e**^*pC*^–the electric field vector direction in the crystal coordinate system–is known^72,73^ and implemented in Hussar software^31^. We will assume that this vector does not change due to nonlinearity. We have verified that, for a “worst case crystal” with linear properties of highly birefringent YVO _4_, and Kerr constant of 10^−18^ m^2^/W characteristic for highly nonlinear ZnSe^64^ illuminated with intensity of 3400 GW/cm^2^–the damage threshold intensity of highly resistant BBO crystal for 25 fs pulses at wavelength of 800 nm^73^, the actual change of ***e***^*e*^ components is around 1%. For more common conditions this error will not exceed the one coming from currently obtainable accuracy of the refractive index measurements–10^−4^ (see refractive index measuremnt references in^73^). Total electric field can, therefore, be decomposed into:

23 $${{\bf{E}}}^{C}={{\bf{E}}}^{pC}+{{\bf{E}}}^{qC}=|{{\bf{E}}}^{p}|{{\bf{e}}}^{pC}+|{{\bf{E}}}^{q}|{{\bf{e}}}^{qC}$$

with *p*≠*q*. Moreover, ${f}_{x}^{p}(\omega ,{k}_{x},{k}_{y})=|{{\bf{E}}}^{p}|/{E}_{x}^{p}=\mathrm{1/}{e}_{x}^{p}$ and corresponding ${f}_{y}^{p}$ is also uniquely defined. Therefore, one can write:

24 $${{\bf{E}}}^{C}={f}_{r}^{p}{E}_{r}^{p}{{\bf{e}}}^{pC}+{f}_{s}^{q}{E}_{s}^{q}{{\bf{e}}}^{qC}$$

with *r*, *s* = *x*, *y*. Then, for a medium with second order nonlinearity characterized by nonlinear suscetibility *χ*^(2)^:

25 $${P^{\prime} }^{NL,p}={{\bf{e}}}^{pC}{{\bf{P}}}^{NL}={{\bf{e}}}^{pC}{\varepsilon }_{0}{\chi }^{\mathrm{(2)}}{{\bf{E}}}^{C}{{\bf{E}}}^{C}={\varepsilon }_{0}{{\bf{e}}}^{pC}{\chi }^{\mathrm{(2)}}({f}_{r}^{p}{E}_{r}^{p}{{\bf{e}}}^{pC}+{f}_{s}^{q}{E}_{s}^{q}{{\bf{e}}}^{qC})({f}_{r}^{p}{E}_{r}^{p}{{\bf{e}}}^{pC}+{f}_{s}^{q}{E}_{s}^{q}{{\bf{e}}}^{qC})$$

Which for SHG (*q* + *q* → *p*) and SFG (*p* + *q* → *p*) becomes:

26 $$=\,{\varepsilon }_{0}{\chi }_{{\rm{deff}}}(\omega ,{k}_{x},{k}_{y}){f}_{s}^{q2}\,{E}_{s}^{q2},\,\,\,\,{\chi }_{{\rm{deff}}}={{\bf{e}}}^{pC}{\chi }^{\mathrm{(2)}}{{\bf{e}}}^{qC}{{\bf{e}}}^{qC}$$

and

27 $$=\,2{\varepsilon }_{0}{\chi }_{{\rm{deff}}}(\omega ,{k}_{x},{k}_{y}){f}_{r}^{p}{f}_{s}^{q}\,{E}_{r}^{p}{E}_{s}^{q},\,{\chi }_{{\rm{deff}}}={{\bf{e}}}^{pC}{\chi }^{\mathrm{(2)}}{{\bf{e}}}^{pC}{{\bf{e}}}^{qC},$$

respectively, where *χ*_eff_(*ω*, *k*_*x*_, *k*_*y*_) represents the effective nonlinear coefficients. We have verified that for a set of 18 nonlinear crystals (including most popular like BBO, BiBO and LBO) if the *x* axis is selected along the polarization vector for the wave propagating exactly along the *z* axis ($\hat{x}\parallel {{\bf{E}}}^{p}({\tilde{\omega }}_{532{\rm{nm}}},{k}_{x}=\mathrm{0,}\,{k}_{y}=\mathrm{0)}$) the deviation of coefficients ${f}_{s}^{p}$ from unity is less than 10^−2^ for Gaussian beams with waist above 1.4 *μ*m at 532 nm (divergence of ~7°) and less than 10^−3^ for beam widths above 4.5 *μ*m (divergence of ~2°). Note, therefore, that in practice it is safe to assume ${f}_{s}^{p}=1$ as current methods of measurements of *χ*^(2)^ (or the experimentalists *d* tensor) give results with accuracy of 5–10% at best^73^.

### Arbitrary Fourier rotation

Here, we describe a convenient way of rotating an arbitrarily shaped pulse without the use of interpolation which is erroneous and time consuming when applied to a 3D case. The inspiration for the method comes from the raster image rotation well known in computer graphics^74^ and its less known implementation with Fourier transform^45^. The method is based on shear operation which can be performed through 1D Fourier transformation of the electric field *E*(*x*, *y*, *ζ*) to a mixed space, multiplication by a phase factor and back transformation to (*x*, *y*, *ζ*). The phase factor has to depend linearly on the Fourier space variable as well as on one of the remaining real variables. Two shear operations are required for single rotations. The definitions of the operators and corresponding Fourier transform operations are listed in the Table 1.

**Table 1:** Shear and scaling operations used for 3D pulse rotation.

name	matrix	operation
*T*_*κ*,*x*_(*a*)	$$(\begin{array}{ccc}1 & 0 & 0\\ 0 & 1 & 0\\ a & 0 & 1\end{array})$$	*F*_*κ*_{*E*(*x*, *y*, *κ*)*e*^*iaκx*^}
${T}_{{k}_{x},z}(d)$	$$(\begin{array}{ccc}1 & 0 & d\\ 0 & 1 & 0\\ 0 & 0 & 1\end{array})$$	${F}_{{k}_{x}}\{E({k}_{x},y,z){e}^{id{k}_{x}z}\}$
*T*_*κ*,*y*_(*h*)	$$(\begin{array}{ccc}1 & 0 & 0\\ 0 & 1 & 0\\ 0 & h & 1\end{array})$$	*F*_*κ*_{*E*(*x*, *y*, *κ*)*e*^*ihκy*^}
${T}_{{k}_{y},z}(j)$	$$(\begin{array}{ccc}1 & 0 & 0\\ 0 & 1 & j\\ 0 & 0 & 1\end{array})$$	${F}_{{k}_{y}}\{E(x,{k}_{y},z){e}^{ij{k}_{y}z}\}$
${T}_{{k}_{y},x}(f)$	$$(\begin{array}{ccc}1 & 0 & 0\\ f & 1 & 0\\ 0 & 0 & 1\end{array})$$	${F}_{{k}_{y}}\{E(x,{k}_{y},z){e}^{if{k}_{y}x}\}$
${T}_{{k}_{x},y}(g)$	$$(\begin{array}{ccc}1 & g & 0\\ 0 & 1 & 0\\ 0 & 0 & 1\end{array})$$	${F}_{{k}_{x}}\{E({k}_{x},y,z){e}^{ig{k}_{x}y}\}$
*S*	$$(\begin{array}{ccc}{S}_{x} & 0 & 0\\ 0 & {S}_{y} & 0\\ 0 & 0 & {S}_{z}\end{array})$$	*E*(*Sxx*, *Syy*, *Szz*)

The second column presents the geometrical shear operation matrices while the third column presents their analytical Fourier transform based counterparts.

A traditional 3D rotation can be constructed from 3 rotations: *R*_*z*_(*φ*)*R*_*y*_(*θ*)*R*_*z*_(*δ*). The three Euler angles corresponding to consecutive rotations around *z*, *y* and *z* axes (by *δ*, *θ* and *φ*, respectively) are used to achieve complete freedom of pulse manipulation. Figure 4 presents an example - a temporal Gaussian pulse with Hermite-Gaussian spatial mode has been propagated in a linear regime after rotation. In our case, however, another construction will also have to be used i.e.: *R*_*z*_(*φ*′)*R*_*x*_(*θ*′)*R*_*z*_(*δ*′) with second rotation performed around *x* axis. Therefore, the above described rotation can be constructed in the following way:

28 $$E^{\prime} (x,y,\zeta )=\mathop{\underbrace{{T}_{{k}_{x},y}({g}_{\phi })\,{T}_{{k}_{y},x}({f}_{\phi })}}\limits_{{R}_{z}(\phi )}\,\,\mathop{\underbrace{{T}_{{k}_{x},z}(d)\,{T}_{{k}_{z},x}(a)}}\limits_{{R}_{y}(\theta )}\,\,\mathop{\underbrace{{T}_{{k}_{x},y}(g)\,{T}_{{k}_{y},x}(f)}}\limits_{{R}_{z}(\delta )}SE(x,y,\zeta )$$

with the parameter values as follows:

29 $$\begin{array}{c}{S}_{x}={({c}_{\theta }{c}_{\varphi }{c}_{\delta })}^{-1},\,{S}_{y}={c}_{\varphi }{c}_{\delta },\,{S}_{z}={c}_{\theta },\,a=-\,{c}_{\theta }{s}_{\theta }{c}_{\varphi },\,d={s}_{\theta }{({c}_{\theta }{c}_{\varphi })}^{-1},\\ f={c}_{\theta }{c}_{\varphi }^{2}{c}_{\delta }{s}_{\delta },\,g=-\,{s}_{\delta }{({c}_{\theta }{c}_{\varphi }^{2}{c}_{\delta })}^{-1},\,{f}_{\phi }={c}_{\varphi }{s}_{\varphi },\,{g}_{\phi }=-{s}_{\varphi }{({c}_{\varphi })}^{-1}\end{array}$$

or

30 $$E^{\prime} (x,y,\zeta )={T}_{{k}_{x},y}({g}_{\phi })\,{T}_{{k}_{y},x}({f}_{\phi })\,\mathop{\underbrace{{T}_{{k}_{z},y}(j)\,{T}_{{k}_{y},z}(h)}}\limits_{{R}_{x}(\theta )}\,{T}_{{k}_{x},y}(g)\,{T}_{{k}_{y},x}(f)\,S\,E(x,y,\zeta )$$

with:

31 $$\begin{array}{c}{S}_{x}={({c}_{\varphi }{c}_{\delta })}^{-1},\,{S}_{y}={c}_{\theta }{c}_{\varphi }{c}_{\delta },\,{S}_{z}={c}_{\theta }^{-1},\,j=-\,{c}_{\theta }{s}_{\theta }{c}_{\varphi },\,h={s}_{\theta }{(\cos \theta {c}_{\phi })}^{-1},\\ f={c}_{\theta }{c}_{\phi }^{2}{c}_{\delta }{s}_{\delta },g=-{s}_{\delta }{({c}_{\theta }{c}_{\phi }^{2}{c}_{\delta }{s}_{\delta })}^{-1},\,{f}_{\phi }={c}_{\varphi }{s}_{\varphi },\,{g}_{\phi }=-{s}_{\varphi }{({c}_{\varphi })}^{-1}\end{array}$$

It is worth to note that the order of the shear operations for each rotation can be reversed (eg.: ${T}_{{k}_{x},y}(g)\,{T}_{{k}_{y},x}(f)\to {T}_{{k}_{y},x}(f^{\prime} )\,{T}_{{k}_{x},y}(g^{\prime} ))$. In such a case, however, the parameters for the shears as well as for the scaling, have to be recalculated. The calculation of the parameters Eqs (29) and (31) have been performed with help of a symbolic Matlab tool. The rotation procedure have been verified, first on the geometrical object (cuboid) with use of matrix operations (see second column in Table 1), then on the 3D matrix representing electric field with use of the Fourier transform operations.

The use of Fourier transform for rotations is limited to around 60° ^45^. Any rotation by angle *α* > 45° can be, however, decomposed into a trivial 90° rotation and rotation by 90° − *α*. Thus, to perform rotations by higher angle values we propose following procedure:

- if *φ* ∈ [0°, 45°] ∩ [315°360°[use standard rotation (second rotation around y axis).
- if *φ* ∈ [45°, 135°] set *φ* → *φ* − 90°, *δ* → *δ* + 90°, *θ* → −*θ*, perform second rotation around x axis.
- if *φ* ∈ [135°, 225°] set *φ* → *φ* − 180°, *θ* → −*θ*, use standard rotation.
- if *φ* ∈ [225°, 315°] set *φ* → *φ* − 270°, *δ* → *δ* + 270°, perform second rotation around x axis.
  possibly, through adding or subtracting 360° bring *δ* back into a [0°, 360°[range, then:
- if *δ* ∈ [0°, 45°] ∩ [315°360°[use *E* − *y*, *x*, *ζ*)
- if *δ* ∈ ]45°, 135°] replace *E*(*x*, *y*, *ζ*) with *E*(−*y*, *x*,*ζ*)
- if *δ* ∈ ]135°, 225°] replace *E*(*x*, *y*, *ζ*) with *E*(−*x*, −*y*, *ζ*)
- if *δ* ∈ ]225°, 315°] replace *E*(*x*, *y*, *ζ*) with *E*(*y*, −*x*, *ζ*)

The scheme presented above can be used for *θ* ∈ [0°, 60°]. For *θ* > 60° a scheme involving 90° rotations in the *x* − *ζ* and *y* − *ζ* plane would be required. Small corner regions of the rotated surface are affected by artifacts arising from the periodic nature of the Fourier transform algorithm^45^. This, however, is a minor concern when the electric field is concentrated in the center of the *x* − *y* − *ζ* plane, which is the usual case with an optical pulse.

The speed advantage of the Fourier transform base rotation with respect to the 3D interpolation (MATLAB’s griddata function) for different grid sizes is presented in Fig. 3. The comparison have been performed on a single “interlagos” class node of the Hydra cluster of the Interdisciplinary Centre for Mathematical and Computational Modelling. For each grid size 10 rotations were performed. For a grid with a size of 4096 × 256 × 256 the rotation through interpolation takes 20 hours, while the same rotation performed with Fourier transform approach takes around 46 seconds (around 1500 times faster).

## Electronic supplementary material

Chirp pulse interference.

Up conversion process.

Four-wave mixing in boxcar configuration.

Supplementary information

## Data Availability

The datasets generated during the current study are available from the corresponding author on reasonable request.

## References

[1] Arisholm Gunnar (1997). General numerical methods for simulating second-order nonlinear interactions in birefringent media. Journal of the Optical Society of America B.

[2] Kolesik, M., Moloney, J. V. & Mlejnek, M. Unidirectional Optical Pulse Propagation Equation. *Phys. Rev. Lett*. **89**, 10.1103/Phys-RevLett.89.283902 (2002). 10.1103/PhysRevLett.89.283902 12513148

[3] Kolesik, M. & Moloney, J. V. Nonlinear optical pulse propagation simulation: From Maxwell’s to unidirectional equations. *Phys. Rev. E* **70**, 10.1103/PhysRevE.70.036604 (2004). 10.1103/PhysRevE.70.036604 15524653

[4] Stanislauskas, T., Balčiūnas, I., Tamuliene, V., Budriūnas, R. & Varanavičius, A. Analysis of parametric fluorescence amplified in a noncollinear optical parametric amplifier pumped by the second harmonic of a femtosecond Yb: KGW laser. *Lith. J. Phys*. **56**, http://www.lmaleidykla.lt/ojs/index.php/physics/article/view/3271 (2016).

[5] Migdał P, Wasilewski W (2010). Noise reduction in 3d noncollinear parametric amplifier. Appl. Phys. B.

[6] Arisholm Gunnar, Bieger Jens, Schlup Philip, Hauri Christoph P., Keller Ursula (2004). Ultra-broadband chirped-pulse optical parametric amplifier with angularly dispersed beams. Optics Express.

[7] Thai Alexandre, Skrobol Christoph, Bates Philip K., Arisholm Gunnar, Major Zsuzsanna, Krausz Ferenc, Karsch Stefan, Biegert Jens (2010). Simulations of petawatt-class few-cycle optical-parametric chirped-pulse amplification, including nonlinear refractive index effects. Optics Letters.

[8] Stepanenko Yuriy, Radzewicz Czesław (2006). Multipass non-collinear optical parametric amplifier for femtosecond pulses. Optics Express.

[9] Wnuk Paweł, Stepanenko Yuriy, Radzewicz Czesław (2009). Multi-terawatt chirped pulse optical parametric amplifier with a time-shear power amplification stage. Optics Express.

[10] Wnuk Paweł, Stepanenko Yuriy, Radzewicz Czesław (2010). High gain broadband amplification of ultraviolet pulses in optical parametric chirped pulse amplifier. Optics Express.

[11] Erny C, Gallmann L, Keller U (2009). High-repetition-rate femtosecond optical parametric chirped-pulse amplifier in the mid-infrared. Appl. Phys. B.

[12] Nishikawa T, Uesugi N (1995). Effects of walk-off and group velocity difference on the optical parametric generation in KTiOPO 4 crystals. J. Appl. Phys..

[13] Pang D, Zhang R, Wang Q (2001). Theoretical analysis of noncollinear phase-matched optical parametric ampliR er seeded by a white-light continuum. Opt. Commun..

[14] Zhang Ruobing, Pang Dongqing, Wang Qingyue (2002). Theoretical analysis of a noncollinear phase-matched optical parametric amplifier seeded by an optical parametric generation. Applied Optics.

[15] Lang T., Harth A., Matyschok J., Binhammer T., Schultze M., Morgner U. (2013). Impact of temporal, spatial and cascaded effects on the pulse formation in ultra-broadband parametric amplifiers. Optics Express.

[16] Hong Z, Zhang Q, Lan P, Lu P (2014). Generation of few-cycle infrared pulses from a degenerate dual-pump OPCPA. Opt. Express.

[17] Charbonneau-Lefort Mathieu, Afeyan Bedros, Fejer M. M. (2010). Theory and simulation of gain-guided noncollinear modes in chirped quasi-phase-matched optical parametric amplifiers. Journal of the Optical Society of America B.

[18] Prandolini MJ (2014). Design considerations for a high power, ultrabroadband optical parametric chirped-pulse amplifier. Opt. Express.

[19] Riedel R (2014). Thermal properties of borate crystals for high power optical parametric chirped-pulse amplification. Opt. Express.

[20] Wilhelm T., Piel J., Riedle E. (1997). Sub-20-fs pulses tunable across the visible from a blue-pumped single-pass noncollinear parametric converter. Optics Letters.

[21] Cerullo G, Nisoli M, De Silvestri S (1997). Generation of 11 fs pulses tunable across the visible by optical parametric amplification. Appl. Phys. Lett..

[22] Shirakawa A, Sakane I, Takasaka M, Kobayashi T (1999). Sub-5-fs visible pulse generation by pulse-front-matched noncollinear optical parametric amplification. Appl. Phys. Lett..

[23] Arisholm Gunnar, Paschotta Rüdiger, Südmeyer Thomas (2004). Limits to the power scalability of high-gain optical parametric amplifiers. Journal of the Optical Society of America B.

[24] Tavella F, Marcinkevičius A, Krausz F (2006). Investigation of the superfluorescence and signal amplification in an ultrabroadband multiterawatt optical parametric chirped pulse amplifier system. New J. Phys..

[25] Herrmann D, Tautz R, Tavella F, Krausz F, Veisz L (2010). Investigation of two-beam-pumped noncollinear optical parametric chirped-pulse amplification for the generation of few-cycle light pulses. Opt. Express.

[26] Guo X (2014). Non-collinear phase-matching geometries in optical parametric chirped-pulse amplification. Opt. Commun..

[27] Picozzi, A. & Haelterman, M. Influence of walk-off, dispersion, and diffraction on the coherence of parametric fluorescence. *Phys. Rev. E* **63**, 10.1103/PhysRevE.63.056611 (2001). 10.1103/PhysRevE.63.056611 11415035

[28] Manzoni Cristian, Moses Jeffrey, Kärtner Franz X., Cerullo Giulio (2011). Excess quantum noise in optical parametric chirped-pulse amplification. Optics Express.

[29] Yoon JW (2012). Improvement of contrast ratio in saturated OPCPA system by using pump pulse shaping and time delay control. Opt. Commun..

[30] Stepanenko Yuriy (2011). On the efficiency of a multiterawatt optical parametric amplifier: numerical model and optimization. Journal of the Optical Society of America B.

[31] Kardaś TM (2017). Full 3d modelling of pulse propagation enables efficient nonlinear frequency conversion with low energy laser pulses in a single-element tripler. Sci. Reports.

[32] Schanz R, Kovalenko SA, Kharlanov V, Ernsting NP (2001). Broad-band fluorescence upconversion for femtosecond spectroscopy. Appl. Phys. Lett..

[33] Sajadi M, Quick M, Ernsting NP (2013). Femtosecond broadband fluorescence spectroscopy by down- and up-conversion in b-barium borate crystals. Appl. Phys. Lett..

[34] Gerecke M, Bierhance G, Gutmann M, Ernsting NP, Rosspeintner A (2016). Femtosecond broadband fluorescence upconversion spectroscopy: Spectral coverage versus efficiency. Rev. Sci. Instruments.

[35] Tulej M, Knopp G, Gerber T, Radi PP (2010). Degenerate and two-color resonant four-wave mixing of *c*_2_ in a molecular beam environment. J. Raman Spectrosc..

[36] Matsuda, Y. & Lee, Y.-P. Two-color resonant four-wave mixing spectroscopy of the X 1 A 1 (500) state of SO 2 in a supersonic jet. *Chem. physics letters* **362**, 235–242, http://www.sciencedirect.com/science/article/pii/S0009261402011004 (2002).

[37] Williams Skip, Rohlfing Eric A., Rahn Larry A., Zare Richard N. (1997). Two-color resonant four-wave mixing: Analytical expressions for signal intensity. The Journal of Chemical Physics.

[38] Fisher KAG (2016). Frequency and bandwidth conversion of single photons in a room-temperature diamond quantum memory. Nat. Commun..

[39] Di Donato M (2015). Identification of the Excited-State C=C and C=O Modes of trans -b-Apo-8′-carotenal with Transient 2d-IR-EXSY and Femtosecond Stimulated Raman Spectroscopy. The J. Phys. Chem. Lett..

[40] Kardaś TM (2014). Dynamics of the time-resolved stimulated Raman scattering spectrum in presence of transient vibronic inversion of population on the example of optically excited trans-b-apo-8′-carotenal. The J. Chem. Phys..

[41] Di Donato M (2014). Combination of Transient 2d-IR Experiments and Ab Initio Computations Sheds Light on the Formation of the Charge-Transfer State in Photoexcited Carbonyl Carotenoids. The J. Phys. Chem. B.

[42] Taflove, A. & Hagness, S. C. *Computational Electrodynamics: The Finite-Difference Time-Domain Method*, second edn (Artech House, Boston, 2000).

[43] Dissanayake CM, Premaratne M, Rukhlenko ID, Agrawal GP (2010). FDTD modeling of anisotropic nonlinear optical phenomena in silicon waveguides. Opt. Express.

[44] Boyd, J. P. *Chebyshev and Fourier Spectral Methods*, second edn (DOVER Publications, Inc., 2000).

[45] Larkin KG, Oldfield MA, Klemm H (1997). Fast fourier method for the accurate rotation of sampled images. Opt. Commun..

[46] Gaeta AL (2000). Catastrophic collapse of ultrashort pulses. Phys. Rev. Lett..

[47] Gulley, J. R. & Dennis, W. M. Ultrashort-pulse propagation through free-carrier plasmas. *Phys. Rev. A* **81**, 10.1103/PhysRevA.81.033818 (2010).

[48] Kolesik M, Moloney JV (2014). Modeling and simulation techniques in extreme nonlinear optics of gaseous and condensed media. Reports on Prog. Phys..

[49] Couairon A (2011). Practitioner’s guide to laser pulse propagation models and simulation: Numerical implementation and practical usage of modern pulse propagation models. The Eur. Phys. J. Special Top..

[50] Smith, A. V. Snlo – nonlinear optics code (2016).

[51] Whalen P, Brio M, Moloney J (2015). Exponential time-differencing with embedded Runge–Kutta adaptive step control. J. Comput. Phys..

[52] Hairer, E., Nørsett, S. P. & Wanner, G. *Solving Ordinary Differential Equations I: Nonstiff Problems*, 2nd ed. edn (springer, New York, 1993).

[53] Hochbruck M, Ostermann A (2010). Exponential integrators. Acta Numer..

[54] Patil S, Takale M, Navare S, Dongare M (2011). Cross focusing of two coaxial cosh-Gaussian laser beams in a parabolic medium. Optik - Int. J. for Light. Electron Opt..

[55] Konar S, Jana S, Mishra M (2005). Induced focusing and all optical switching in cubic quintic nonlinear media. Opt. Commun..

[56] Boyraz, Ö., Koonath, P., Raghunathan, V. & Jalali, B. All optical switching and continuum generation in silicon waveguides. *Opt. Express* **12**, 4094–4102, https://www.osapublishing.org/abstract.cfm?uri=oe-12-17-4094 (2004). 10.1364/opex.12.004094 19483951

[57] Sharping J, Fiorentino M, Kumar P, Windeler R (2002). All-optical switching based on cross-phase modulation in microstructure fiber. IEEE Photonics Technol. Lett..

[58] Melloni A., Chinello M., Martinelli M. (2000). All-optical switching in phase-shifted fiber Bragg grating. IEEE Photonics Technology Letters.

[59] Wu, J., Cai, H., Peng, Y. & Zeng, H. Controllable supercontinuum generation by the quantum wake of molecular alignment. *Phys. Rev. A* **79**, 10.1103/PhysRevA.79.041404 (2009).

[60] Gupta MK, Sharma RP, Gupta VL (2005). Cross focusing of two laser beams and plasma wave excitation. Phys. Plasmas.

[61] Sodha MS, Mishra SK, Agarwal SK (2007). Self-focusing and cross-focusing of Gaussian electromagnetic beams in fully ionized collisional magnetoplasmas. Phys. Plasmas.

[62] Sharma RP, Chauhan PK (2008). Nonparaxial theory of cross-focusing of two laser beams and its effects on plasma wave excitation and particle acceleration: Relativistic case. Phys. Plasmas.

[63] Białkowski B, Stepanenko Y, Nejbauer M, Radzewicz C, Waluk J (2012). The dynamics and origin of the unrelaxed fluorescence of free-base tetraphenylporphyrin. J. Photochem. Photobiol. A: Chem..

[64] Major, A., Aitchison, J. S., Smith, P. W. E., Sorokin, E. & Sorokina, I. T. Z-scan characterization of the nonlinear refractive index of single crystal ZnSe in the 1.20–1.95 mm wavelength range. In Morandotti, R. A., Ruda, H. E. & Yao, J. (eds) *Proc. of SPIE, 59710H*, 10.1117/12.628686 (SPIE, 2005).

[65] Salem Reza, Foster Mark A., Turner Amy C., Geraghty David F., Lipson Michal, Gaeta Alexander L. (2008). Optical time lens based on four-wave mixing on a silicon chip. Optics Letters.

[66] Salem R, Foster MA, Gaeta AL (2013). Application of space–time duality to ultrahigh-speed optical signal processing. Adv. Opt. Photonics.

[67] Agrawal, G. *Nonlinear fiber optics* (Academic press, 2001).

[68] Brabec T, Krausz F (1997). Nonlinear optical pulse propagation in the single-cycle regime. Phys. Rev. Lett..

[69] Cole, I. R. *Modelling CPV*. Ph.D. thesis, Loughborough University, https://dspace.lboro.ac.uk/dspace-jspui/handle/2134/18050 (2015).

[70] Eberly, D. Euler angle formulas. Tech. Rep., Geometric Tools, LLC, http://www.geometrictools.com/ (1999).

[71] Born, M. & Wolf, E. *Principles of optics*, 7th edn (Cambridge University Press, Cambridge, 1999).

[72] Roberts D.A. (1992). Simplified characterization of uniaxial and biaxial nonlinear optical crystals: a plea for standardization of nomenclature and conventions. IEEE Journal of Quantum Electronics.

[73] Dmitriev, V. G., Gurzadyan, G. G. & Nikogosyan, D. N. *Handbook of Nonlinear Optical Crystals, vol. 64 of Springer series in optical sciences*, third edn (Springer, 1999).

[74] Paeth A (1986). A fast algorithm for general raster rotation. Graph. Interface’.

