# Supplementary material for: Noncollinear and nonlinear pulse propagation
Source: Sci Rep. 2018 Sep 25;8:14350. doi: 10.1038/s41598-018-32676-9 (PMC6156600; doi:10.1038/s41598-018-32676-9)
Supplement: Supplementary file 4 — Supplementary information [file 41598_2018_32676_MOESM4_ESM.pdf]

Noncollinear and nonlinear pulse propagation – supplementary information

Tomasz M. Kardaś, Yuriy Stepanenko and Czesław Radzewicz

July 8, 2018

Method tests

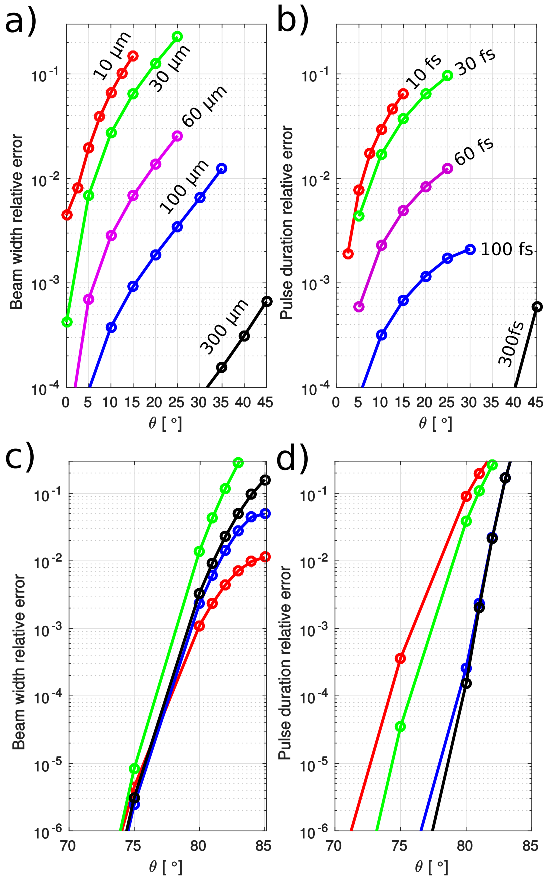

Figure 1: Relative error of beam width (a,c) and pulse duration (b, d) after propagation in 5 mm of BBO crystal as a ordinary polarization. θ indicates the angle between the beam propagation direction and the model's axis. The approximated solution method (a,b) and the rotated UPPE method (c,d). Results have been obtained for five pulses with following parameters: (i) 10 fs (duration FWHM), 10 μm (waist) – red, (ii) 30 fs, 30 μm – green, (iii) 60 fs, 60 μm – magenta, (iv) 100 fs, 100 μm – blue and (v) 300 fs, 300 μm – black.

One way of approximating the noncollinear propagation is to use pulses with shifted spatial spectra, and the standard (not rotated) UPPE [1, 5]. In order to quantify errors coming from this approximation a series of simulations with different pulse durations and spatial sizes have been performed. Fig. 1 presents relative error of the values of the beam width and pulse duration obtained from the simulation of propagation through 5 mm of BBO crystal with respect to the theoretical values.

Fig. 1(a,b) presents results obtained for the approximated case. It is apparent that one has to limit himself to 5° to keep the error bellow 2% for 10 μm beams, and to 10° to keep the error bellow 3% for 30 μm. For larger beams the acceptable angle range increases. The limitation for the pulse duration is also apparent. Angles not higher than 7.5° and 10° can be used for 10 fs and 30 fs pulses if the error bellow 2% is desired.

Fig. 1(c,d) presents results obtained with rotated UPPE approach. In case of rotated UPPE method the ultimate limitation appears when pulse contains components that would propagate in the negative z' axis direction. These components cannot be propagated with a propagation like model and, thus, the error must grow near the 90° limit. Apparently even for the shortest and most divergent beams it possible to perform simulations with both relative errors below 10^{-6} for angles up to 70° (or, for a two beam simulation, mutual angle of 140°). For the angle range bellow 65°the errors drop down below the level of 10^{-11} (not shown).

Linear propagation simulations were obtained through single step of Exponential Euler method [2] with grid sizes of 8192(t) x 4096(x) while the spatial and temporal dimensions were adjusted (0.6 – 6 mm and 1–54 ps) to best fit the simulated pulses.

Another set of test included simulations for extremely short and focused pulses. The short pulse central wavelength of 256 nm (cycle: 0.8 fs) was chosen to fit within the center of validity of Sellmeier formula for fused silica. Gaussian pulses with beam width of 10 μm and durations of: 1.15 (1.4 cycle), 3, 10, 30 and 100 fs were propagated through 1 mm of fused silica. A grid optimized for shortest pulse was used. It contained 16384(t) x 4096(x) points spanning 6.7 ps in time and 0.5 mm in space. Fig. 2. presents spatial intensity distribution of the 1.15, 3 and 10 fs pulses after propagation performed colinearly and with 60° deviation from z axis direction. The initially shortest pulse becomes the longest due to refractive index dispersion. Fig. 4. presents the pulse duration errors for pulses propagating at different angles relative to the duration of the pulse propagating colinearly with z axis. All errors are below 3×10^{-3}. The errors for 10, 30 and 100 fs pulses are higher then these for 3 fs pulse. This comes from the fact that the grid is suboptimal for longer pulses.

A 10 fs pulses at 800 nm were used for study of focused beam propagation. Gaussian beams with widths of 1 (19° divergence), 3, 10 and 30 μm were simulated. A grid with 8192(t) x 4096(x) points spanning 3.4 ps in time and 1 mm in space was used. Fig. 3. presents spatial intensity distribution of the 1 and 3 μm pulses after propagation colinearly and with 60° deviation from z axis direction through 1 mm of fused silica. The curvature of the wavefront of the highly divergent pulse is clearly visible. Fig. 4. presents the beam width errors relative to the width of the pulse propagating colinearly with z axis for pulses propagating at different angles. For the 1 μm beam errors are bellow 3×10^{-6} while for less divergent beams they are bellow 2×10^{-13}. This suggests that the error for 1 μm beam could be further reduced through increase of resolution.

A collinear (coaxial) second harmonic generation (SHG) process is a perfect choice for nonlinear propagation tests against the existing collinear simulation codes. SHG of the 800 nm pulses in the BBO crystal were used as the test case here. Slight deviation from perfect phase-matching was chosen. The results of forward propagation ($\theta = 0°$) have been compared with SNLO [4] and Hussar software [3] and found to be in perfect agreement. To test the fidelity of our rotation method the simulations with the pulse propagating noncollinearly with the simulation box were also performed. Since the angle between the input beam's wavevector and the crystal's optic axis was fixed a perfect simulation platform should return results identical to those from collinear simulation.

In correspondence to linear propagation the tests were performed for both rotated UPPE and the approximate situation where non-rotated UPPE is used. Fig. 5(a–c) presents the relative energy, beam width and pulse duration errors for different beam sizes, pulse durations and energies when the approximation of non-rotated UPPE version is used. Fig. 5(d–f) show the same errors for rotated UPPE. Apparently even for the smallest beam sizes accurate simulations (global error below 10^{-3}) for noncollinearity angles as high as 70° can be performed with use of the rotated UPPE. This is not the case for the approximated method, where angles below 5° have to be used for smallest beams and pulse durations in order to maintain the error level.

References

[1] Gunnar Arisholm, Jens Biegert, Philip Schlup, Christoph Hauri, and Ursula Keller. Ultra-broadband chirped-pulse optical parametric amplifier with angularly dispersed beams. *Optics express*, 12(3):518–530, 2004.

[2] Marlis Hochbruck and Alexander Ostermann. Exponential integrators. *Acta Numerica*, 19:209–286, May 2010.

[3] Tomasz M. Kardaś, Micha Nejbauer, Pawe Wnuk, Bojan Resan, Czesaw Radzewicz, and Piotr Wasylczyk. Full 3d modelling of pulse propagation enables efficient nonlinear frequency conversion with low energy laser pulses in a single-element tripler. *Scientific Reports*, 7:42889, February 2017.

[4] A. V. Smith. Snlo – nonlinear optics code, 2016.

[5] Alexandre Thai, Christoph Skrobol, Philip K. Bates, Gunnar Arisholm, Zsuzsanna Major, Ferenc Krausz, Stefan Karsch, and Jens Biegert. Simulations of petawatt-class few-cycle optical-parametric chirped-pulse amplification, including nonlinear refractive index effects. *Optics letters*, 35(20):3471–3473, 2010.

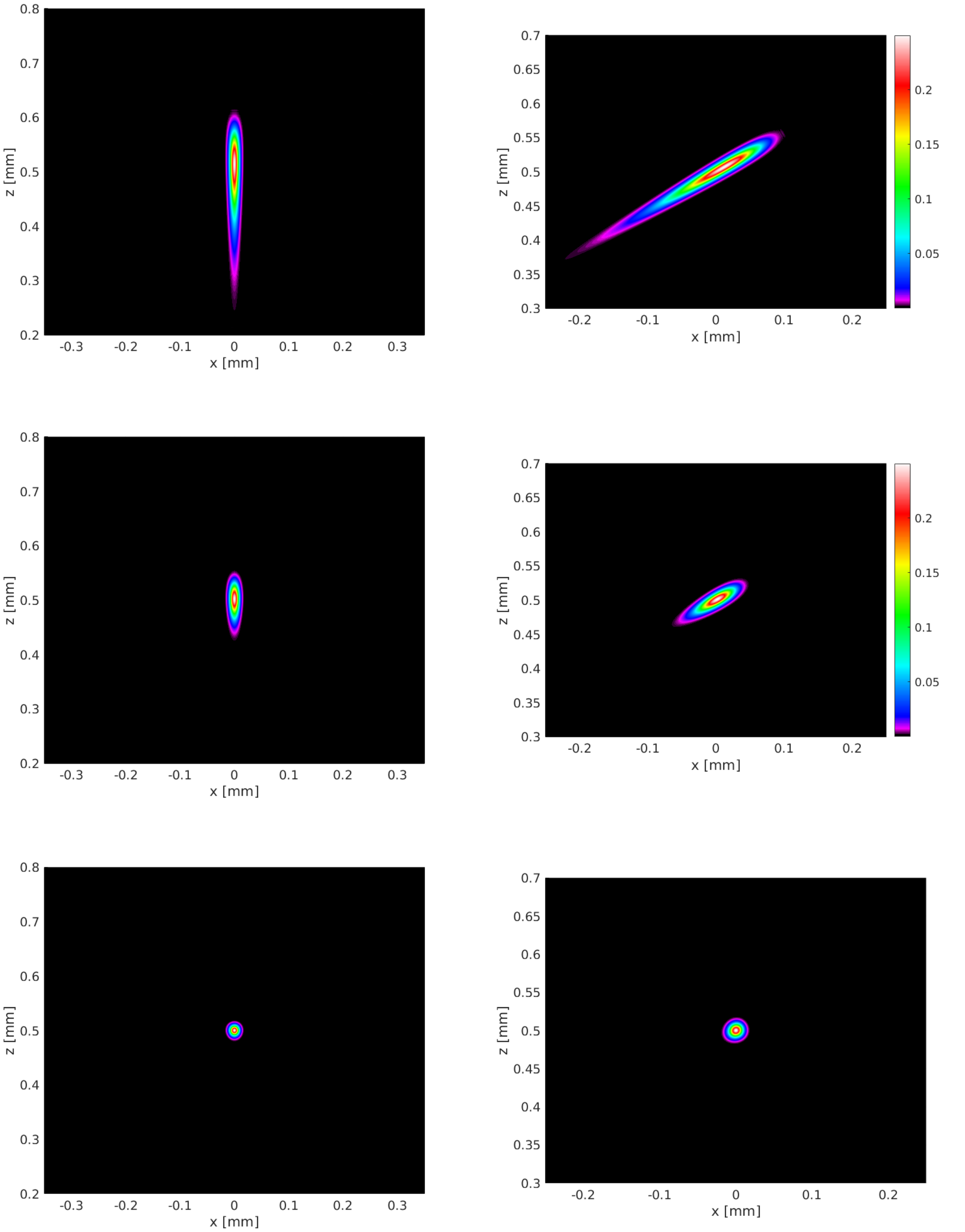

Figure 2: Results of propagation in 1 mm of fused silica of Gaussian pulses with beam widths of 10 μm and durations of 1.15, 3, 10 fs, from top to bottom respectively. The results of collinear (left) and noncoliner (60° – right) simulation are presented.

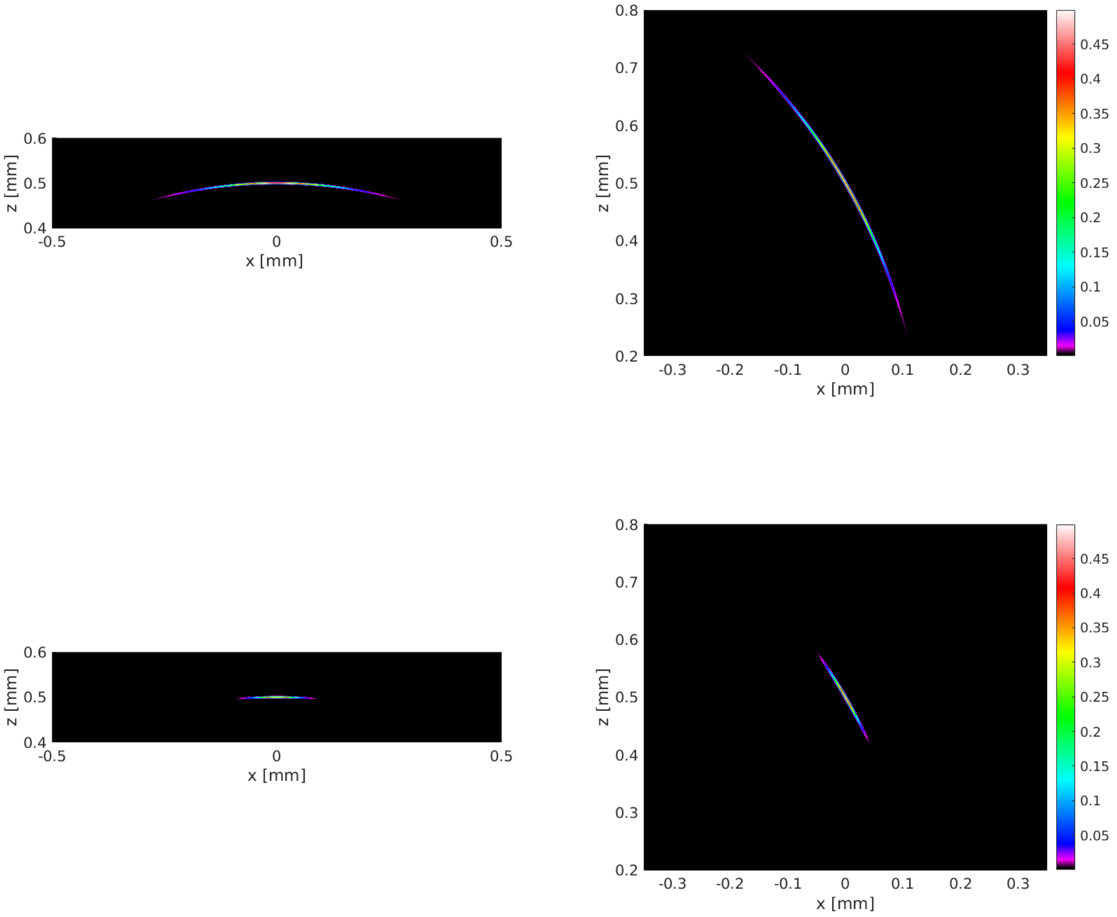

Figure 3: Results of propagation in 1 mm of fused silica of 10 fs Gaussian pulses with widths of 1 and 3 μm (top and bottom, respectively). The results of collinear (left) and noncoliner (60° – right) simulation are presented.

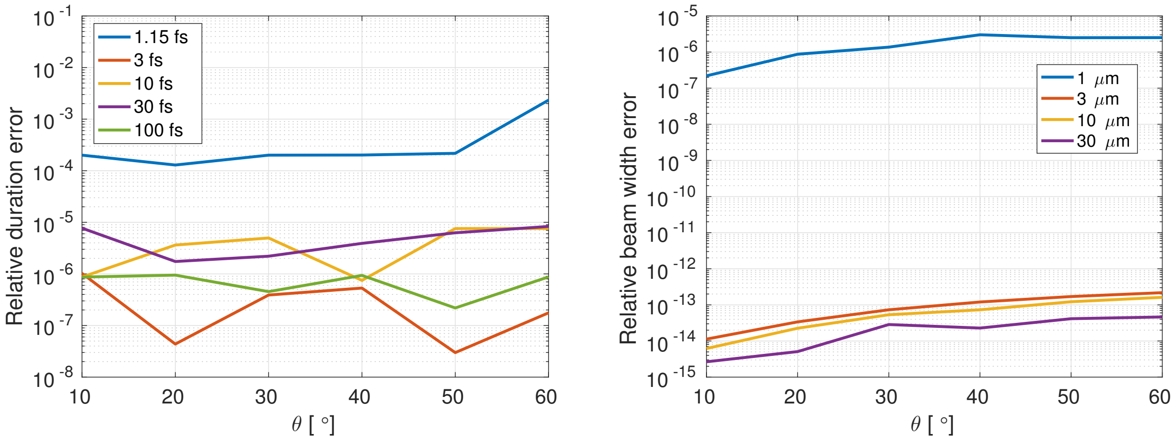

Figure 4: Propagation angle dependent pulse duration and beam width errors with respect to the collinear simulation case.

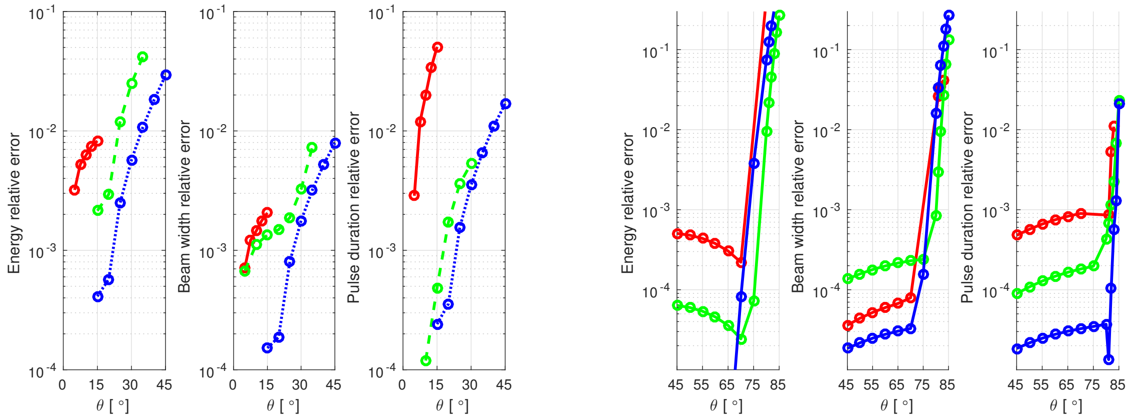

Figure 5: Relative errors of the pulse energy, beam width and pulse duration after SHG in 5 mm of BBO crystal for the approximated method (three plots on the left) and acurate method (three plots on the right). Input pulses 10 fs (temporal FWHM), 100 μm (spatial beam waist) 0.2 nJ pulse energy (red), 100 fs, 100 μm, 30 nJ (green) and 1 ps, 1 mm, 1 μJ (blue).
